# High-intensity interval training modulates APP expression and reduces neuronal apoptosis in a β-amyloid-induced model of Alzheimer’s disease: hippocampal evidence

**DOI:** 10.1007/s00221-026-07397-5

**Published:** 2026-09-25

**Authors:** Welton Daniel Nogueira Godinho, Francisco Sérgio Lopes Vasconcelos Filho, Cícero Matheus Lima Amaral, Maria Izabel Florindo Guedes, Guilherme Lisboa de Serpa, Adriano Cesar Carneiro Loureiro, Vânia Marilande Ceccatto, Paula Matias Soares

**Affiliations:** 1 Laboratório de Bioquímica E Expressão Gênica, Instituto Superior de Ciências Biomédicas, Universidade Estadual Do Ceará, Av. Dr. Silas Munguba, 1700, Itaperi, Fortaleza, CE 60714-903 Brazil; 2 Coordenadoria de Esporte E Cultura Do Movimento, Universidade Federal Do Cariri, Av. Ten. Raimundo Rocha, 1639, Cidade Universitária, Juazeiro Do Norte, CE 63048-080 Brazil; 3 Laboratório de Biotecnologia E Biologia Molecular, Biotecnologia Em Saúde, Universidade Estadual Do Ceará, Av. Dr. Silas Munguba, 1700, Itaperi, Fortaleza, CE 60714-903 Brazil; 4 Laboratório de Fisiologia Endócrina E Metabolismo, Universidade Estadual Do Ceará, Av. Dr. Silas Munguba, 1700, Itaperi, Fortaleza, CE 60714-903 Brazil

**Keywords:** Alzheimer’s disease, HIIT, APP, FNDC5, PPARGC1A, Neuroprotection

## Abstract

High-intensity interval training (HIIT) has emerged as a promising non-pharmacological strategy for neuroprotection, although its molecular effects in Alzheimer’s disease (AD) remain unclear. This study investigated the effects of HIIT on gene expression (FNDC5, PPARGC1A, APP), protein expression, cognitive performance, and neuronal apoptosis in a β-amyloid-induced rat model of AD. Sixty male Wistar rats were divided into four groups: sham sedentary (SS), sham trained (ST), Alzheimer sedentary (AS), and Alzheimer trained (AT). AD-like pathology was induced by intrahippocampal infusion of β-amyloid (Aβ1–42). Animals underwent an 8-week HIIT protocol. Gene expression was assessed by qPCR using the ΔCt method, and statistical analyses were performed using two-way ANOVA or mixed-design ANOVA followed by Tukey’s post hoc test. HIIT significantly increased PPARGC1A expression (*p* < 0.05), while FNDC5 gene expression remained unchanged (*p* > 0.05). However, FNDC5 protein expression was increased in trained groups. APP gene expression was significantly reduced at the transcriptional level (*p* < 0.05), whereas APP protein abundance differed among experimental conditions without a uniform training-related reduction. HIIT improved physical performance (*p* < 0.0001) and reduced hippocampal neuronal apoptosis (*p* < 0.05). In the Morris water maze, improvement was observed only within the Alzheimer-trained group, with no significant differences between groups. HIIT was associated with molecular and cellular adaptations consistent with a neuroprotective response in this β-amyloid-induced model, including metabolic-pathway modulation, reduced APP transcription, and attenuated neuronal apoptosis. These effects were not accompanied by robust between-group cognitive improvement.

**Graphical abstract:**

**Figure:**
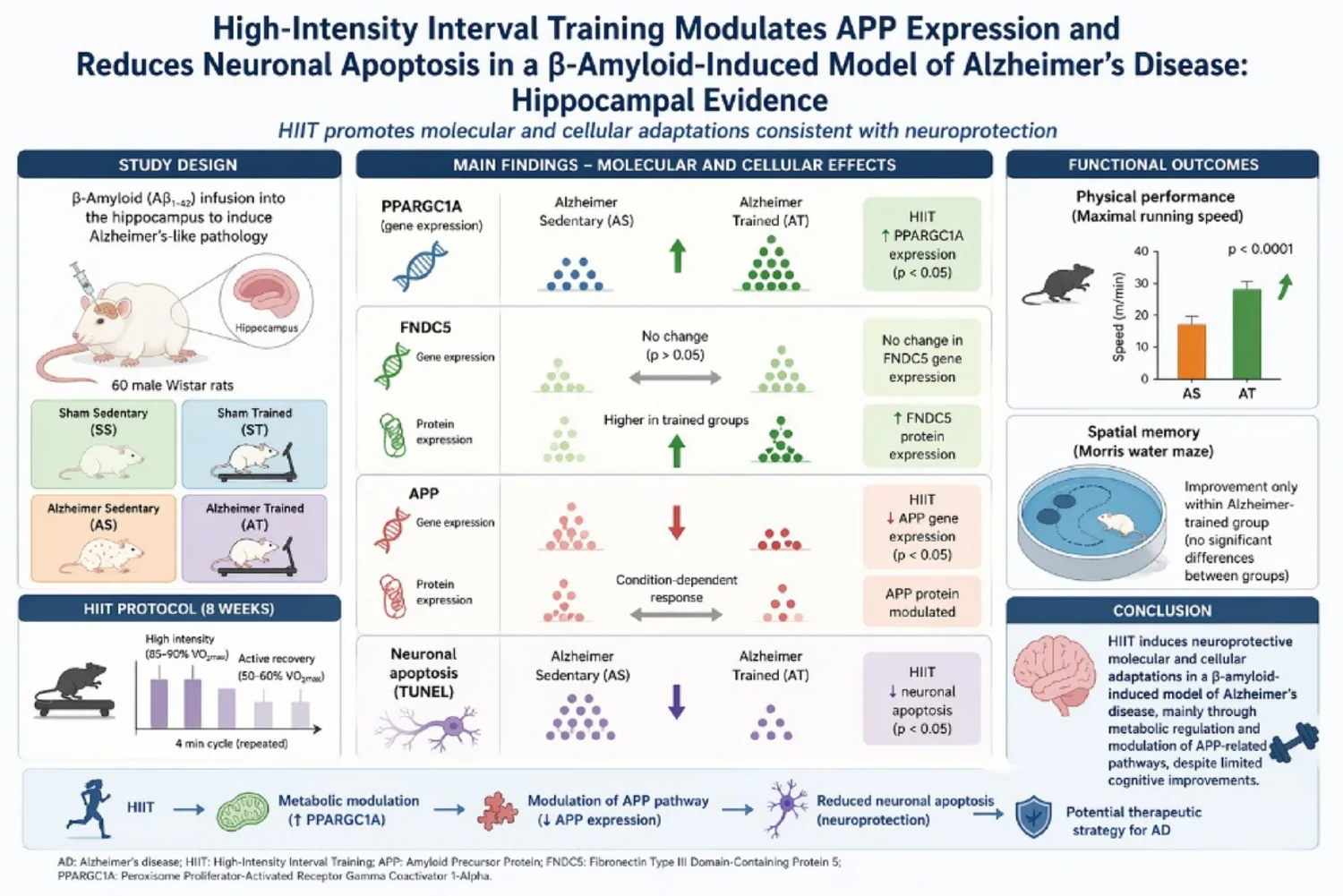

## Introduction

Alzheimer's disease (AD) is the most common cause of dementia worldwide and represents one of the greatest public health challenges of the twenty-first century due to its increasing prevalence, progressive clinical course, and substantial socioeconomic burden. Population aging has markedly increased the number of affected individuals, with current estimates indicating that more than 55 million people worldwide are living with dementia, a figure expected to nearly triple by 2050 if effective disease-modifying interventions are not established. Beyond the progressive decline in memory and other cognitive domains, AD profoundly compromises functional independence and quality of life while imposing considerable emotional and financial burdens on patients, caregivers, and healthcare systems (Alzheimer's Association, 2025; World Health Organization 2050).

Recent advances have substantially changed the conceptual framework of AD. Rather than being defined exclusively by clinical manifestations, the disease is now recognized as a biological continuum characterized by progressive neuropathological alterations that begin years or even decades before the onset of cognitive symptoms. The National Institute on Aging–Alzheimer's Association (NIA-AA) research framework introduced a biomarker-based classification centered on amyloid deposition (A), tau pathology (T), and neurodegeneration (N), emphasizing that molecular alterations precede overt cognitive impairment and highlighting the importance of identifying therapeutic strategies capable of interfering with early pathogenic events (Jack et al. 2018).

The pathological hallmarks of AD include extracellular deposition of amyloid-β (Aβ) peptides, intracellular accumulation of hyperphosphorylated tau protein, chronic neuroinflammation, synaptic dysfunction, and progressive neuronal loss. Among these mechanisms, abnormal processing of the amyloid precursor protein (APP) remains a central event in disease initiation and progression. Sequential cleavage of APP by β- and γ-secretases generates neurotoxic Aβ peptides, particularly Aβ_42_, which exhibit a marked propensity for aggregation into soluble oligomers and extracellular plaques. Increasing evidence indicates that soluble Aβ oligomers are more closely associated with synaptic dysfunction and cognitive decline than amyloid plaque burden itself, suggesting that early molecular changes play a fundamental role in disease progression (Chen et al. 2017; Selkoe and Hardy 2016).

Although the amyloid cascade hypothesis continues to provide an important framework for understanding AD pathogenesis, it is now widely recognized that disease progression results from the interaction of multiple biological processes rather than from isolated amyloid accumulation. Mitochondrial dysfunction, oxidative stress, impaired calcium homeostasis, chronic neuroinflammation, and progressive metabolic failure contribute synergistically to neuronal degeneration. In this context, mitochondrial abnormalities have emerged as one of the earliest detectable events in AD, preceding extensive neuronal loss and amplifying oxidative damage, impaired ATP production, and activation of apoptotic pathways that further compromise synaptic integrity and cognitive function (Liegro et al. 2023; Swerdlow 2023; Tiwari et al. 2019).

The limited clinical efficacy of currently available pharmacological therapies reinforces the need to identify interventions capable of targeting multiple pathogenic pathways simultaneously. Although recently approved anti-amyloid therapies represent important advances, their clinical benefits remain modest and are frequently accompanied by high costs, restricted indications, and safety concerns. Consequently, increasing attention has been directed toward non-pharmacological strategies capable of modulating disease progression through complementary molecular mechanisms. Among these, physical exercise has emerged as one of the most promising interventions because of its broad effects on systemic metabolism, mitochondrial function, neuroplasticity, neuroinflammation, and neuronal survival, supporting its potential role as a disease-modifying strategy in Alzheimer's disease (Cummings et al. 2025; Bertram et al. 2010; Liegro et al. 2023; Knopman et al. 2021).

The central role of amyloid precursor protein (APP) in Alzheimer's disease extends beyond its pathological processing into amyloid-β peptides. APP is a highly conserved transmembrane glycoprotein widely expressed throughout the central nervous system, where it participates in neuronal development, synaptogenesis, axonal growth, intracellular signaling, and maintenance of synaptic plasticity. Under physiological conditions, APP is predominantly processed through the non-amyloidogenic pathway by α-secretase, generating soluble APPα (sAPPα), a neuroprotective fragment associated with neuronal survival and synaptic function. In contrast, cleavage by β-secretase (BACE1), followed by γ-secretase, initiates the amyloidogenic pathway, producing Aβ peptides that progressively accumulate as soluble oligomers and extracellular plaques. This imbalance between physiological and pathological APP processing is considered a pivotal event in AD pathogenesis and contributes directly to synaptic dysfunction, mitochondrial impairment, oxidative stress, and neuronal apoptosis (Selkoe and Hardy 2016; Müller et al. 2017; Dawkins and Small 2014; Wang et al. 2020; Cenini and Voos 2019).

Among the multiple downstream consequences of abnormal APP metabolism, mitochondrial dysfunction has emerged as one of the earliest and most influential mechanisms contributing to neurodegeneration. Experimental and clinical evidence indicates that mitochondrial abnormalities occur before extensive neuronal loss and are characterized by impaired oxidative phosphorylation, excessive production of reactive oxygen species (ROS), reduced ATP synthesis, altered calcium homeostasis, and activation of intrinsic apoptotic pathways. These metabolic disturbances establish a vicious cycle in which oxidative damage further enhances amyloidogenic APP processing while Aβ accumulation exacerbates mitochondrial dysfunction, ultimately accelerating synaptic degeneration and cognitive decline. Consequently, interventions capable of restoring mitochondrial homeostasis have become attractive therapeutic targets in AD research (Tiwari et al. 2019; Wang et al. 2020; Cenini and Voos 2019; Lin and Beal 2006; Swerdlow 2018; Liegro et al. 2023; Erickson et al. 2019).

Growing evidence suggests that physical exercise represents one of the most effective non-pharmacological interventions capable of simultaneously modulating several of these pathogenic mechanisms. In addition to its well-established cardiovascular and metabolic benefits, regular exercise promotes structural and functional adaptations within the central nervous system, including enhanced cerebral perfusion, improved mitochondrial function, increased neurogenesis, reduced neuroinflammation, stimulation of synaptic plasticity, and preservation of cognitive performance. These neuroprotective effects are mediated not only by systemic metabolic adaptations but also by the release of exercise-induced signaling molecules that establish a dynamic communication between skeletal muscle and the brain, reinforcing the concept of the muscle–brain axis (Cotman and Berchtold 2002; Pedersen 2011; Pedersen and Febbraio 2012; Kobilo et al. 2011; Puigserver and Spiegelman 2003).

Among the molecular mediators activated by exercise, peroxisome proliferator-activated receptor gamma coactivator-1 alpha (PGC-1α), encoded by the PPARGC1A gene, occupies a central position in the regulation of mitochondrial biogenesis and cellular energy metabolism. PGC-1α coordinates the transcription of numerous genes involved in oxidative phosphorylation, antioxidant defense, fatty acid oxidation, and mitochondrial quality control, thereby increasing the resistance of neurons to metabolic stress. Importantly, experimental studies have demonstrated that increased PGC-1α activity suppresses BACE1 expression, reducing amyloidogenic APP processing and subsequent Aβ production, suggesting that metabolic adaptations induced by exercise may directly influence one of the principal pathogenic pathways of AD (Handschin and Spiegelman 2008; St-Pierre et al. 2006; Katsouri et al. 2011; Boström et al. 2012).

Activation of PGC-1α also induces expression of fibronectin type III domain-containing protein 5 (FNDC5), a membrane protein that is cleaved to generate the myokine irisin during physical exercise. Initially recognized for its role in systemic energy metabolism, the PGC-1α/FNDC5/irisin signaling axis has more recently been implicated in brain function and neuroprotection. Experimental evidence demonstrates that irisin crosses or signals across the blood–brain interface, enhances hippocampal expression of brain-derived neurotrophic factor (BDNF), promotes synaptic plasticity, stimulates neurogenesis, attenuates neuroinflammation, and improves learning and memory in animal models. Furthermore, reduced circulating and cerebral levels of FNDC5/irisin have been reported in patients with Alzheimer's disease, reinforcing the hypothesis that impairment of this pathway may contribute to disease progression (Wrann et al. 2013; Lourenco et al. 2019; Kim et al. 2018; Islam et al. 2024; Falck et al. 2024; Paxinos and Watson 2014).

Collectively, these findings support the concept that exercise-induced activation of the PGC-1α/FNDC5 signaling pathway may integrate mitochondrial adaptation, regulation of APP metabolism, attenuation of neuronal apoptosis, and preservation of hippocampal function. Nevertheless, despite the growing body of evidence supporting the neuroprotective effects of exercise, the molecular interactions linking these pathways remain incompletely understood, particularly in the context of high-intensity interval training (HIIT), which has emerged as a potent stimulus for mitochondrial remodeling and metabolic adaptation.

Among the different exercise modalities, high-intensity interval training (HIIT) has attracted considerable attention because it elicits robust metabolic and mitochondrial adaptations while requiring a relatively low training volume. Compared with moderate-intensity continuous training, HIIT has been shown to produce greater improvements in mitochondrial biogenesis, oxidative capacity, insulin sensitivity, and cardiovascular fitness. Emerging evidence also suggests that HIIT enhances hippocampal plasticity, attenuates neuroinflammation, increases neurotrophic factor expression, and improves cognitive performance in experimental models of neurodegenerative diseases. These characteristics make HIIT a promising non-pharmacological strategy for targeting multiple pathogenic mechanisms involved in AD simultaneously (Paxinos and Watson 2014; Long and Holtzman 2019; Strooper and Karran 2016; Hampel et al. 2021; Querfurth and LaFerla 2010; Mattson 2012; Gomez-Pinilla and Hillman 2013; Voss et al. 2013; Sleiman et al. 2016; Wrann 2015; Lourenco and Frozza 2023; Pedersen 2019; Swerdlow 2007; Hardy and Selkoe 2002). Despite these advances, important mechanistic questions remain unresolved. Although previous studies have independently demonstrated beneficial effects of exercise on APP metabolism, mitochondrial function, or FNDC5/irisin signaling, few investigations have simultaneously evaluated these pathways within the same experimental model. In particular, the relationship between HIIT-induced metabolic adaptations and the coordinated modulation of PPARGC1A**,** FNDC5**,** ITGB5**,** APP expression, neuronal apoptosis, and cognitive performance has not been comprehensively investigated. Understanding these interactions may provide important insights into the molecular mechanisms through which exercise delays or attenuates the progression of AD and may contribute to the identification of novel therapeutic targets based on endogenous neuroprotective pathways (Boström et al. 2012; Wrann et al. 2013; Lourenco et al. 2019; Kim et al. 2018; Islam et al. 2024; Falck et al. 2024; Paxinos and Watson 2014, 1997; Voss et al. 2013; Sleiman et al. 2016; Wrann 2015; Lourenco and Frozza 2023; Pedersen 2019; Swerdlow 2007; Hardy and Selkoe 2002; Wilson et al. 2023).

Based on these considerations, we hypothesized that HIIT would be associated with reduced hippocampal neuronal apoptosis and with changes in PPARGC1A, FNDC5, ITGB5, and APP expression, together with improved cognitive performance in a β-amyloid-induced rat model. Because the study did not experimentally manipulate these molecular targets, their coordinated assessment was intended to identify associations rather than establish a causal signaling sequence.

Therefore, the aim of the present study was to investigate the effects of an eight-week HIIT protocol on spatial learning and memory, hippocampal gene expression (PPARGC1A**,** FNDC5**,** ITGB5**,** and APP), and neuronal apoptosis in a β-amyloid-induced rat model of Alzheimer's disease. By integrating behavioral, molecular, and histological outcomes, this study sought to clarify the mechanisms underlying exercise-induced neuroprotection and to further explore the therapeutic potential of HIIT as a multimodal intervention for Alzheimer's disease.

## Objectives

The objective of this study was to investigate the effects of HIIT on the hippocampal expression of FNDC5, ITGB5, PPARGC1A, and APP, protein expression, spatial memory, physical performance, and neuronal apoptosis in a β-amyloid-induced model of Alzheimer’s disease.

## Materials and methods

### Animals

Sixty male Wistar rats (3–4 months old; 250–300 g) were used in this study. The animals were housed under controlled conditions (24 ± 2 °C, 12 h light–dark cycle) with free access to food and water.

At the end of the experimental period, animals were anesthetized with ketamine (100 mg/kg) and xylazine (10 mg/kg) and euthanized by decapitation for collection of the hippocampus and other tissues of interest.

All experimental procedures were conducted in accordance with international guidelines for the care and use of laboratory animals and were approved by the Animal Ethics Committee of the State University of Ceará (protocol no. 08526890/2020). All animal procedures, including stereotaxic surgery, exercise interventions, behavioral assessments, and tissue collection, were completed within the validity period of the approved protocol. Every effort was made to minimize animal suffering and reduce the number of animals used. Subsequent laboratory analyses, statistical analyses, and manuscript preparation were performed after completion of the animal experiments and did not involve additional animal procedures (Kilkenny et al. 2010; Percie du Sert et al. 2020).

### Experimental design

Animals were randomly assigned to four experimental groups (n = 15 per group): sham sedentary (SS), sham trained (ST), Alzheimer sedentary (AS), and Alzheimer trained (AT).

Each group was subdivided for specific analyses: five animals for gene expression, five for histological and immunohistochemical analyses, and five for protein quantification.

Following a 7-day recovery period after surgical procedures, animals underwent behavioral testing and treadmill adaptation. A maximal running test was performed every two weeks to adjust training intensity. In the final week, behavioral tests were conducted prior to training sessions.

The HIIT protocol was performed five days per week for 8 weeks.

### Experimental induction of Alzheimer’s model

Alzheimer-like pathology was induced by stereotaxic injection of β-amyloid (Aβ1–42). The peptide was prepared from a stock solution (1 μg/μL in saline, pH 7.4), incubated at 37 °C for 72 h to allow aggregation, and stored at − 70 °C until use.

Animals were anesthetized with ketamine (100 mg/kg) and xylazine (10 mg/kg, intraperitoneal) and positioned in a stereotaxic apparatus. A midline incision was performed to expose the skull and locate the bregma.

The Aβ aggregate was bilaterally injected into the brain using a 27-gauge needle coupled to a microsyringe at the following coordinates: AP: 0.8 mm; ML: 1.5 mm; DV: 3.6 mm (Tiwari et al. 2019). The infusion rate was 1 μL per 3 min, and the needle was maintained in position for 5 min after injection to allow diffusion before slow withdrawal. The stereotaxic coordinates used correspond to the dorsal hippocampus according to the rat brain atlas of Paxinos and Watson (Zhang et al. 2015).

After surgery, animals received subcutaneous saline (1 mL) to prevent dehydration and were monitored during recovery.

**Fig. 1:**
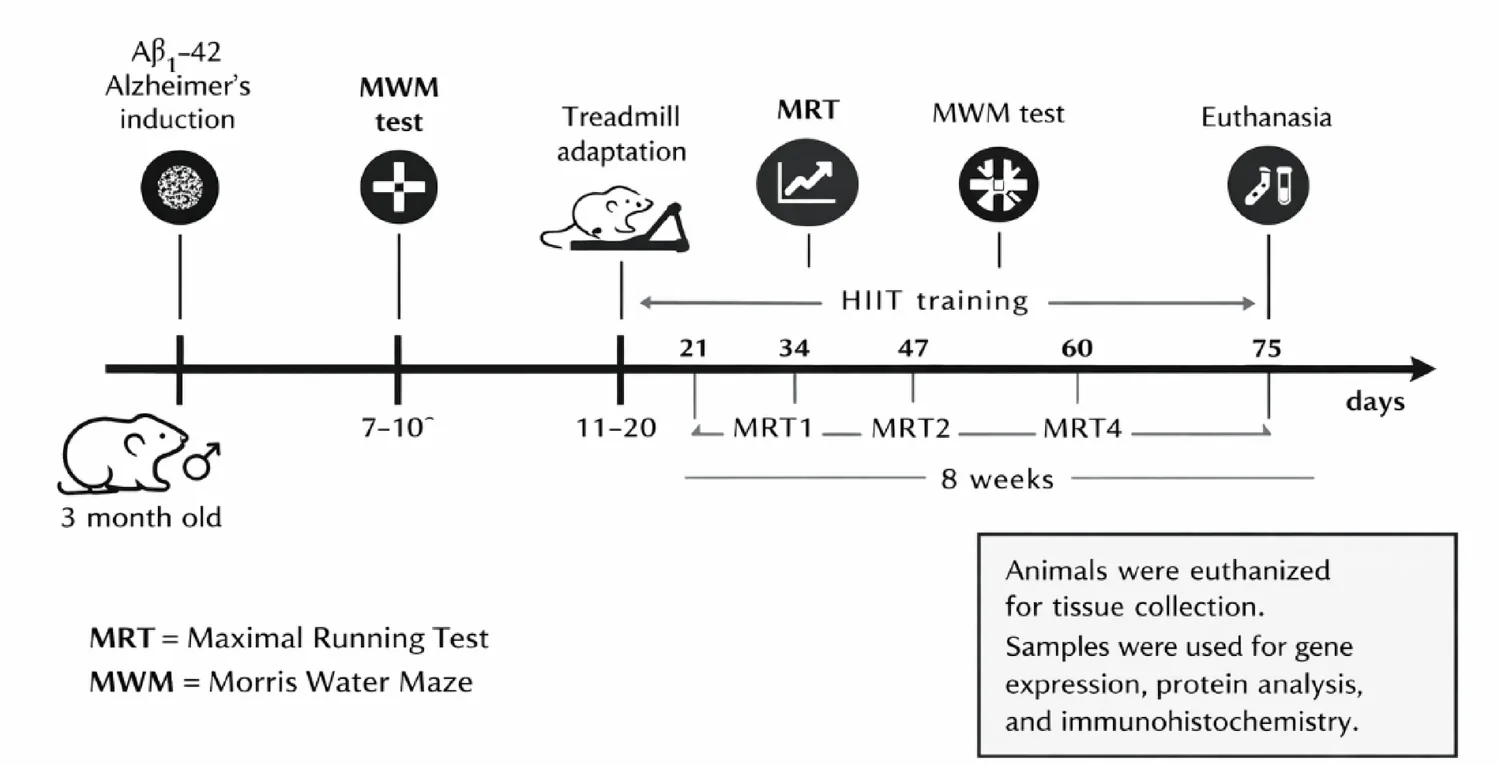
Experimental timeline of the HIIT protocol in a β-amyloid-induced Alzheimer’s model. Wistar rats underwent β-amyloid (Aβ1–42) induction followed by recovery, behavioral testing (MWM), treadmill adaptation, and high-intensity interval training (HIIT) for 8 weeks. Maximal running tests (MRT) were performed periodically to adjust training intensity. Behavioral assessment was repeated after the intervention, followed by euthanasia and tissue collection for molecular and histological analyses. MRT = Maximal Running Test; MWM = Morris Water Maze

Animals were then housed for 7 days to allow recovery and development of pathology, followed by behavioral testing prior to euthanasia (Fig. 1).

### Training protocol

Prior to the training intervention, animals underwent a treadmill adaptation period to minimize stress associated with the experimental procedure. During this phase, rats were exposed to treadmill running for 5–10 min per day at low speeds ranging from 0.4 to 0.8 km/h (Paxinos and Watson 2007).

**Fig. 2:**
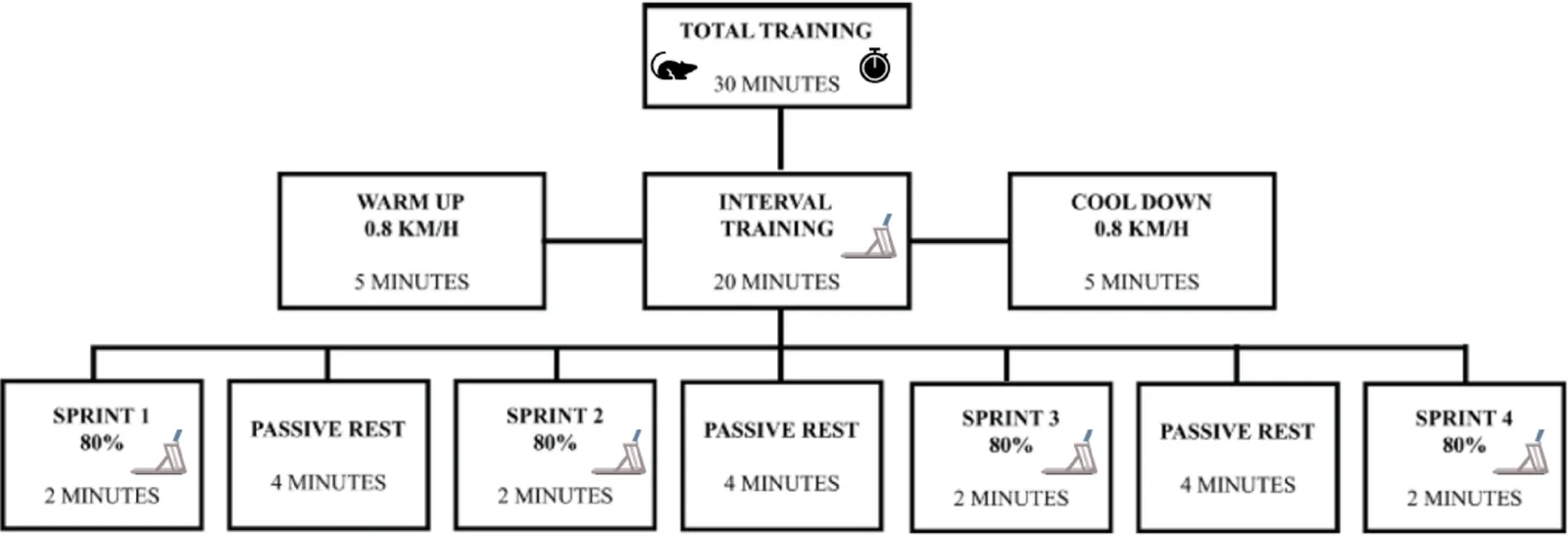
High-intensity interval training (HIIT) protocol applied to Wistar rats. Each training session lasted 30 min and consisted of a 5-min warm-up (0.8 km/h), followed by four high-intensity running bouts (2 min each at 80% of maximal running capacity), interspersed with 4-min passive recovery periods. The session ended with a 5-min cool-down (0.8 km/h)

After the adaptation period, animals assigned to the trained groups (ST and AT) performed a high-intensity interval training (HIIT) protocol, while sedentary groups (SS and AS) were placed on the treadmill for the same duration without running (Fig. 2).

Each training session began with a 5-min warm-up at 0.8 km/h, followed by four high-intensity running bouts lasting 2 min each, performed at 80% of the maximal running capacity determined by the maximal running test (MRT). Each bout was interspersed with a 4-min passive recovery period, resulting in a 2:1 recovery-to-work ratio (Kregel).

After the final interval, a 5-min cool-down period at 0.8 km/h was performed. Training sessions were conducted five days per week for a total duration of 8 weeks.

**Fig. 3:**
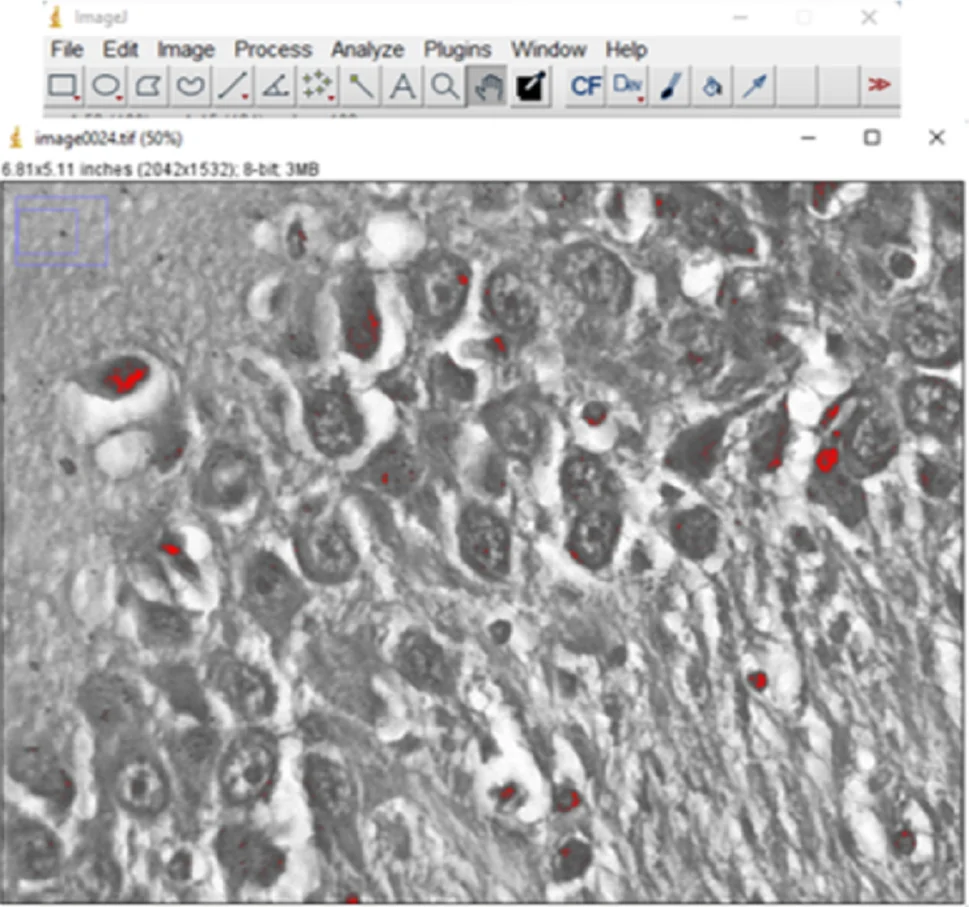
ImageJ workflow used for neuronal quantification in hippocampal sections

The intensity of the exercise was adjusted throughout the protocol based on periodic maximal running tests to ensure maintenance of the relative training load (Fig. 3) (Díaz-Herrera et al. 2001).

### RNA extraction and quantification

Total RNA extraction was performed from hippocampal tissue using TRIzol® reagent (Thermo Fisher Scientific) and the RNeasy Plus Mini Kit® (QIAGEN, Hilden, Germany). RNA integrity was assessed using agarose gel electrophoresis and the Agilent 2100 Bioanalyzer system (Agilent Technologies, Santa Clara, CA, USA), with RNA quality determined by the RNA Integrity Number (RIN) (Bustin et al. 2009).

Gene expression analysis was performed by quantitative real-time PCR (qPCR) using a CFX96 Real-Time PCR Detection System (Bio-Rad, CA, USA). Reactions were performed in duplicate using 2 μL of previously synthesized cDNA, 10 μL of Power SYBR Green Master Mix (Life Technologies, Foster City, CA, USA), 6.8 μL of nuclease-free water, and 0.6 μL (300 nM) each of forward and reverse primers. β-Actin (ACTB) was used as the endogenous reference gene for normalization. Relative gene expression was calculated using the comparative Ct (2^−ΔΔCt) method, with expression levels normalized to β-actin and expressed relative to the Sedentary Sham (SS) group (Table 1).

**Table 1:** Primer sequences used for RT-qPCR analysis

Primer	Sequence (5′–3′)
Β-ACTIN (ACTB)-F	5′ TGTCACCAACTGGGACGATA 3′
Β-ACTIN (ACTB)-R	5′ GGGGTGTTGAAGGTCTCAAA 3′
ITGB5-F	5′ GGAATGCTCGCTGCTTCACC 3′
ITGB5-R	5′ AAGCACAGCTTCCTGGTCGT 3′
FNDC5-F	5′ ACGTGAGGCCGAGAAGATGG 3′
FNDC5-R	5′ TACTGGCGGCAGAAGAGAGC 3′
APP-F	5′ CAACGAGAGGCAGCAGCTTG 3′
APP-R	5′ TAATTCTCGAGGGCCAGGCG 3′
PPARGC1A-F	5′ AGTCCTTCCTCCATGCCTG 3′
PPARGC1A-R	5′ TTGCCATCCCGTAGTTCAC 3′

### Histological analysis

After anesthesia, five animals from each group were transcardially perfused with 50 mM phosphate-buffered saline (PBS), followed by 4% paraformaldehyde in 0.1 M sodium phosphate buffer at pH 7.4.

The brains were removed, dehydrated in ethanol, cleared in xylene, and embedded in paraffin. Coronal sections with a thickness of 6 μm were cut using a microtome (Leica 820, Germany). On average, eight sections were selected from each brain and two sections from each skeletal muscle, following a widely adopted practice for tissue classification, which involves staining with a combination of an acidic and a basic dye. The most common combination worldwide is hematoxylin (a basic dye that imparts a light or dark blue color) and eosin (an acidic dye that imparts a light or dark red color), known as H&E staining, which was used in this experiment.

**Fig. 4:**
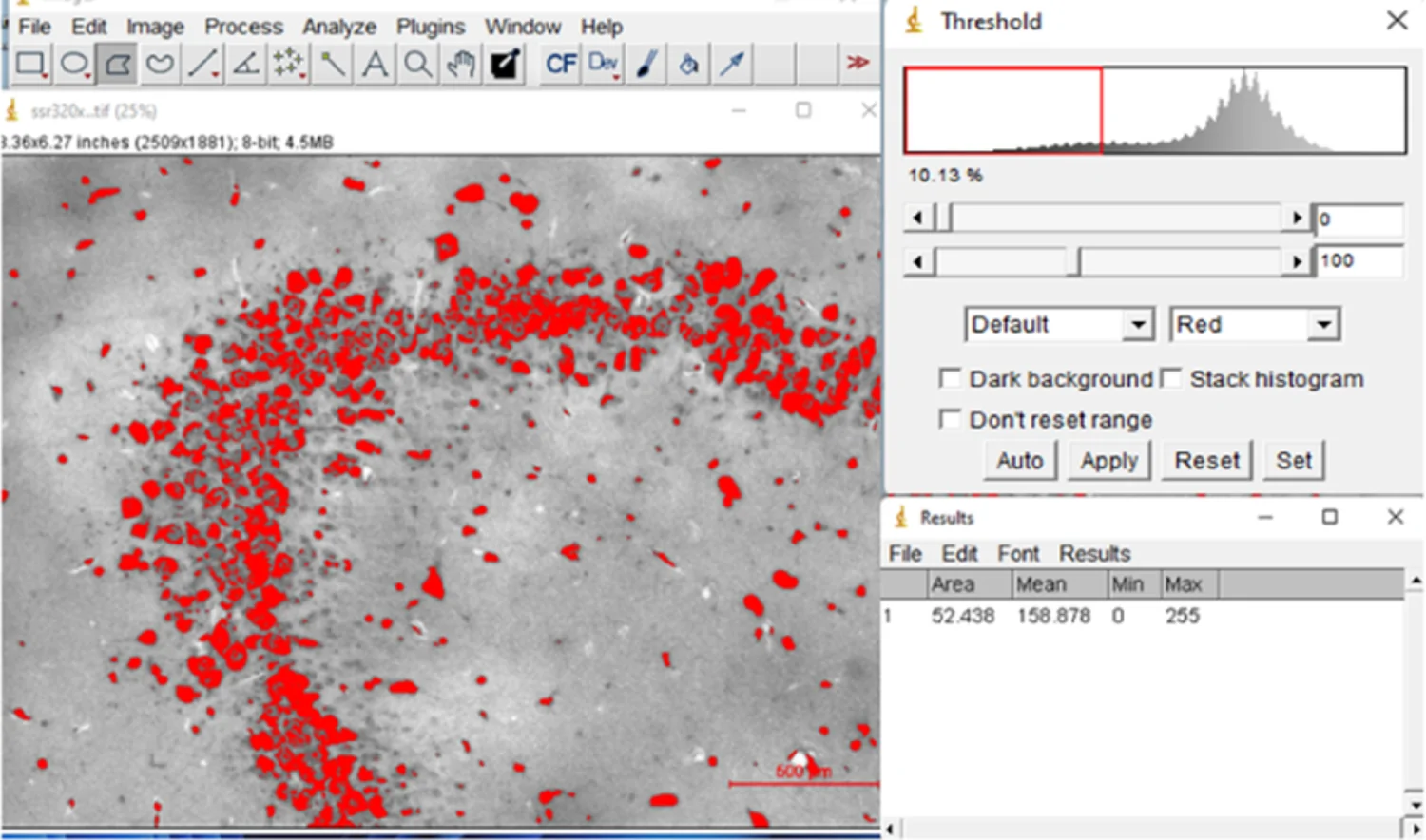
Thresholding procedure used for apoptotic cell quantification with ImageJ (Schindelin et al. 2012)

For analysis of the hippocampal tissue, two fields per histological section were photographed at 4×, 10×, and 20× magnification (brain) and 10× and 20× magnification (muscle). Subsequently, the count of apoptotic neural cells was performed on 10 slides per animal in the hippocampal region using Image J® software (Kobilo et al. 2011). The determination of the histological aspect of neural death followed the criteria established by Klas Blomgren (Liang et al. 2007) (Fig. 4).

### Immunohistochemistry for FNDC5 Detection

**Fig. 5:**
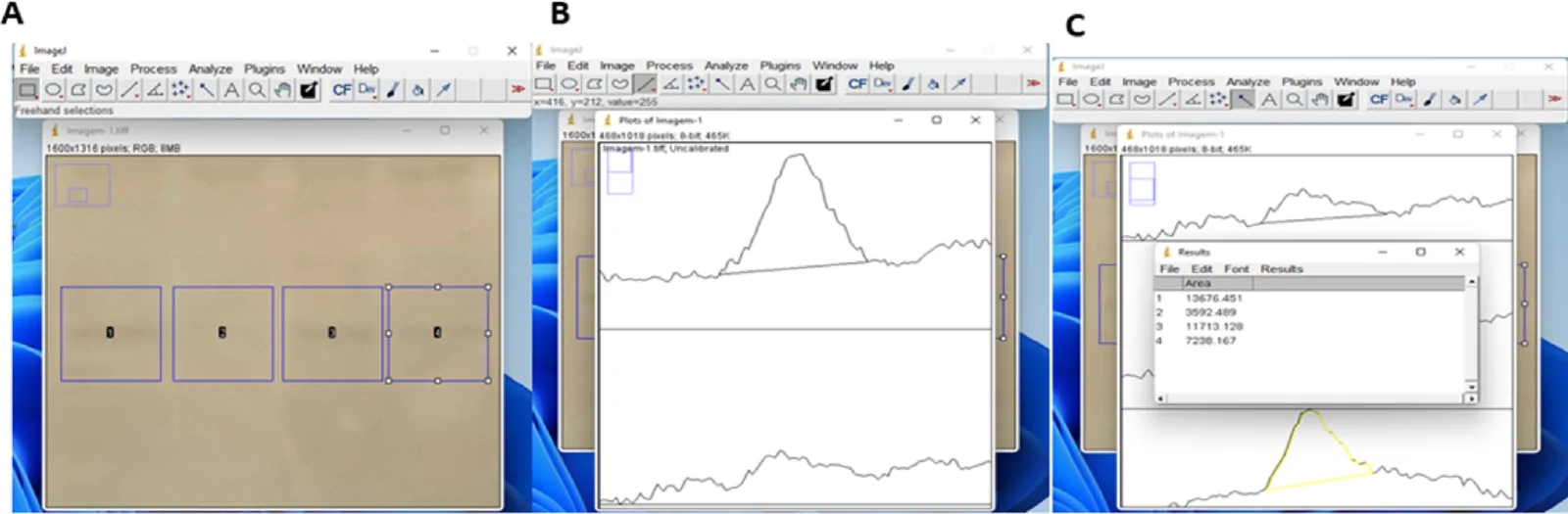
Workflow for Western blot densitometric analysis using ImageJ. Legend: **A** selection of the areas of the bands, **B** Area formatting and perimeter determination, **C** Quantification of the aerials, in the detail in yellow, the space calculated

Immunohistochemistry for FNDC5 in the hippocampus was performed using the streptavidin–biotin-peroxidase method (Blomgren et al. 2003). The FNDC5 Antibody Monoclonal Mouse IgG2B Clone #936,434 Catalog Number: MAB9420 from R&D Systems was used. EnVision™ FLEX+ Mouse (LINKER) was used as the buffered solution containing a stabilizing protein and an antimicrobial agent. It consists of dextran linked to peroxidase molecules and secondary goat antibodies against rabbit and rat immunoglobulins to label membrane proteins. EnVision™ FLEX DAB+ Chromogen, 3,3'-diaminobenzidine tetrahydrochloride in an organic solvent, was used as the chromogen, producing a color ranging from intense violet to colorless without affecting the kit's performance. All reagents were from Dako Agilent (Fig. 5).

Images were captured using a light microscope coupled with a camera and the LAZ 3.5 acquisition system (Leica DM1000, Germany). Three fields per histological section were photographed at 4×, 10×, and 20× magnification. The count of positively labeled cells was performed for each field using Image J® software. Positively labeled cells were considered those showing blue membranous staining. The brain, with a focus on the dentate gyrus of the hippocampus, were marked according to the research interest.

The quantification in the hippocampus was performed using the Image J® software to obtain the total marked area in pixels using the histogram function. This value was recorded in pixels, and then the % Marked Area + was calculated as follows: (% Marked Area + = (area positive for the protein of interest in pixels) × 100 / total area in pixels (converted with an equivalence of 1.244 pixels – 1 µm). This calculation was done for each animal/group, and the Fischer test was applied for comparisons between groups. For the counting of labeled hippocampal cells, the Image J@ software was used, following the methodology described by (Oliveira et al. 2010; Hsu et al. 1982).

### Western blotting/APP

Hippocampal samples, previously dissected and stored at − 80 °C, were homogenized using a Polytron Kinematica® homogenizer in phosphate buffer (Na_2_HPO_4_ 140 mM, KH_2_PO_4_ 130 mM, KCl 50 mM, NaCl 150 mM, pH 7.4) containing 1% aprotinin. Homogenates were centrifuged at 12,000 × g for 15 min at 4 °C (HEMRLE Z365K refrigerated centrifuge), and total protein concentration was determined using the Bradford method (Bradford 1976).

Aliquots containing 20 μg of total protein were mixed with sample buffer (10 mM Tris–HCl, 1 mM EDTA, 0.5% SDS, 1% β-mercaptoethanol, and 0.25% bromophenol blue), heated at 95 °C for 5 min, cooled to room temperature for 5 min, and separated by 12% SDS-PAGE using a Mini-PROTEAN® electrophoresis system (Bio-Rad). Proteins were subsequently transferred onto nitrocellulose membranes, which were blocked overnight at 4 °C with 5% non-fat skim milk.

After three washes with PBS containing 0.05% Tween-20 (10 min each), membranes were incubated with rabbit anti-amyloid precursor protein (APP) antibody (Sigma-Aldrich, A8717; 1:2500) for 1 h at 37 °C, followed by incubation with horseradish peroxidase-conjugated goat anti-rabbit IgG secondary antibody (Sigma-Aldrich, A6154; 1:2500) for 1 h at 37 °C. β-Actin was used as the loading control for normalization of APP protein expression.

After washing, immunoreactive bands were developed using 3,3′-diaminobenzidine (DAB; Sigma-Aldrich, D8001) and nickel chloride (VETEC, CAS 7791–20-0), and images were acquired using a ChemiDoc XRS imaging system (Bio-Rad) under optimized exposure conditions to avoid signal saturation. Band intensities were quantified using ImageJ software, and APP protein expression was normalized to β-actin. Representative blots from two animals per group are presented in the following order: Sedentary Sham (SS), Sedentary Alzheimer's (AS), Trained Sham (ST), and Trained Alzheimer's (AT).

### Statistical analysis

Data normality was assessed using the Shapiro–Wilk test prior to inferential analyses. All data are presented as mean ± standard error of the mean (SEM). Statistical significance was set at *p* < 0.05 (Motulsky 2022).

Gene expression data obtained by quantitative real-time PCR (qPCR) were analyzed using the comparative Ct (2−ΔΔCt) method (Ct_target – Ct_reference), with β-actin used as the reference gene. Relative comparisons between groups were analyzed using two-way analysis of variance (two-way ANOVA), considering two independent factors: pathological condition (SHAM vs. ALZ) and training status (SED vs. TREI). When appropriate, Tukey’s post hoc test was applied for multiple comparisons (Ruxton and Beauchamp 2008).

Protein expression data were also analyzed using two-way ANOVA with the same factors (pathological condition × training), followed by Tukey’s post hoc test when significant main effects or interactions were detected.

Spatial memory performance in the Morris water maze was analyzed using a mixed-design ANOVA (mixed ANOVA), with group (SS, ST, AS, AT) as the between-subjects factor and time (pre-surgery, post-surgery, post-training) as the within-subjects factor. When appropriate, post hoc comparisons were conducted using Tukey’s test.

Data from the maximal exercise test were analyzed using two-way ANOVA, considering group and time as factors, followed by Tukey’s post hoc test.

For histological analyses, including apoptotic neuron quantification, data were analyzed using two-way ANOVA (pathological condition × training), followed by Tukey’s post hoc test.

All analyses were performed using GraphPad Prism software version 9.0 (GraphPad Software, San Diego, CA, USA).

## Results

### Validation of the experimental model

Successful establishment of the experimental model and effectiveness of the HIIT intervention were confirmed through behavioral assessment in the Morris Water Maze and periodic Maximal Running Tests.

### Morris water maze

**Fig. 6:**
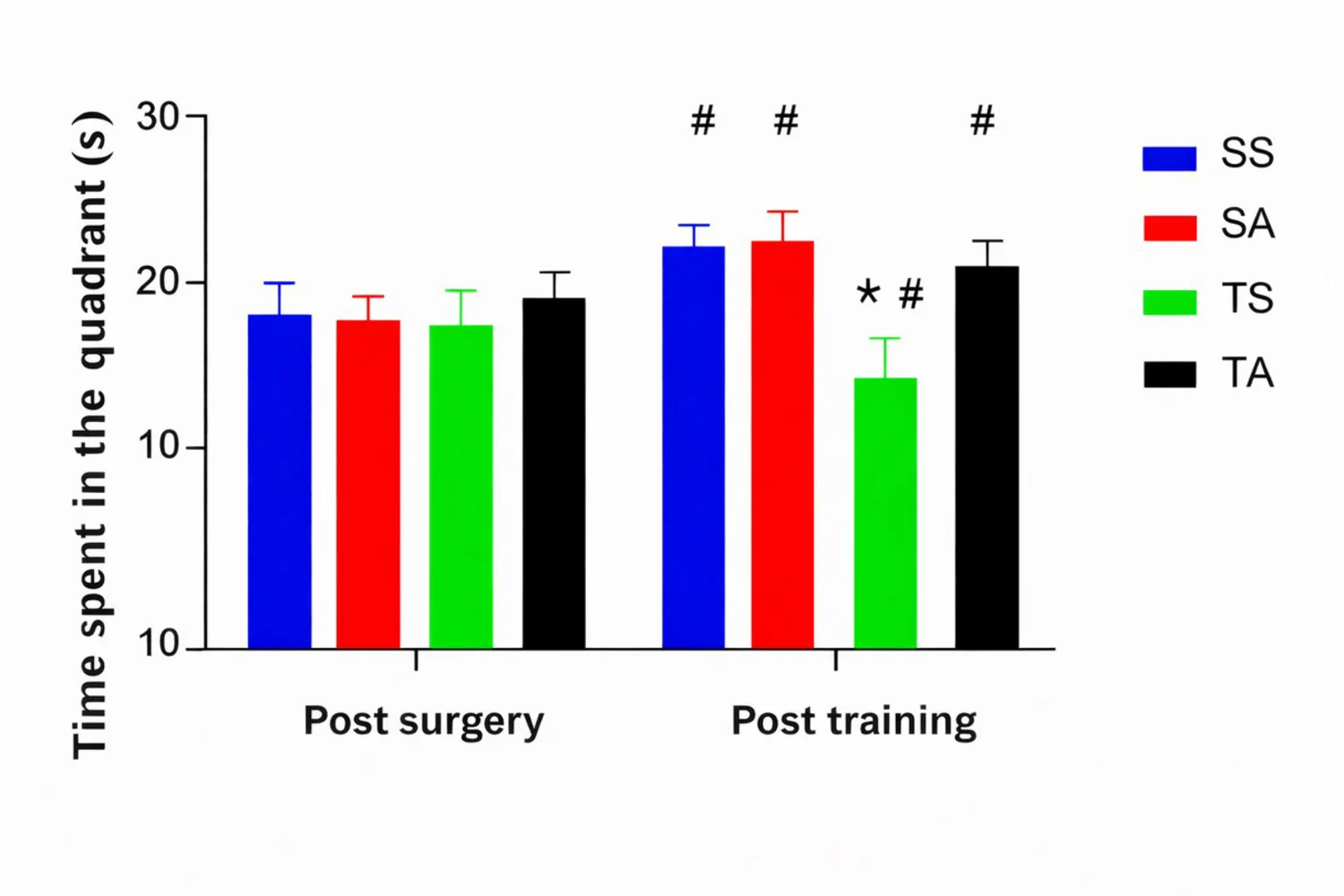
Spatial memory performance in the Morris water maze. Time spent in the target quadrant (seconds) was measured after surgery and after completion of the training protocol in sham sedentary (SS), Alzheimer sedentary (AS), sham trained (ST), and Alzheimer trained (AT) groups. Data are presented as mean ± SEM. Mixed-design ANOVA (group × time) was used for statistical analysis. #* p* < 0.05 vs. post-surgery within the same group; * p < 0.05 vs. SS at the same time point. SS = sham sedentary; AS = Alzheimer sedentary; ST = sham trained; AT = Alzheimer trained

Behavioral performance in the Morris Water Maze was evaluated before β-amyloid infusion, after surgical induction, and following completion of the HIIT protocol (Fig. 6).

Following the 8-week intervention, the Alzheimer-trained group showed significant within-group improvement compared with the post-surgery evaluation; however, no significant differences were observed between experimental groups.

The absence of significant between-group differences indicates that the Morris water maze did not demonstrate a robust cognitive deficit under the present experimental conditions. Accordingly, the behavioral findings cannot, by themselves, confirm the establishment of an Alzheimer-like cognitive phenotype.

### Maximal running test

**Fig. 7:**
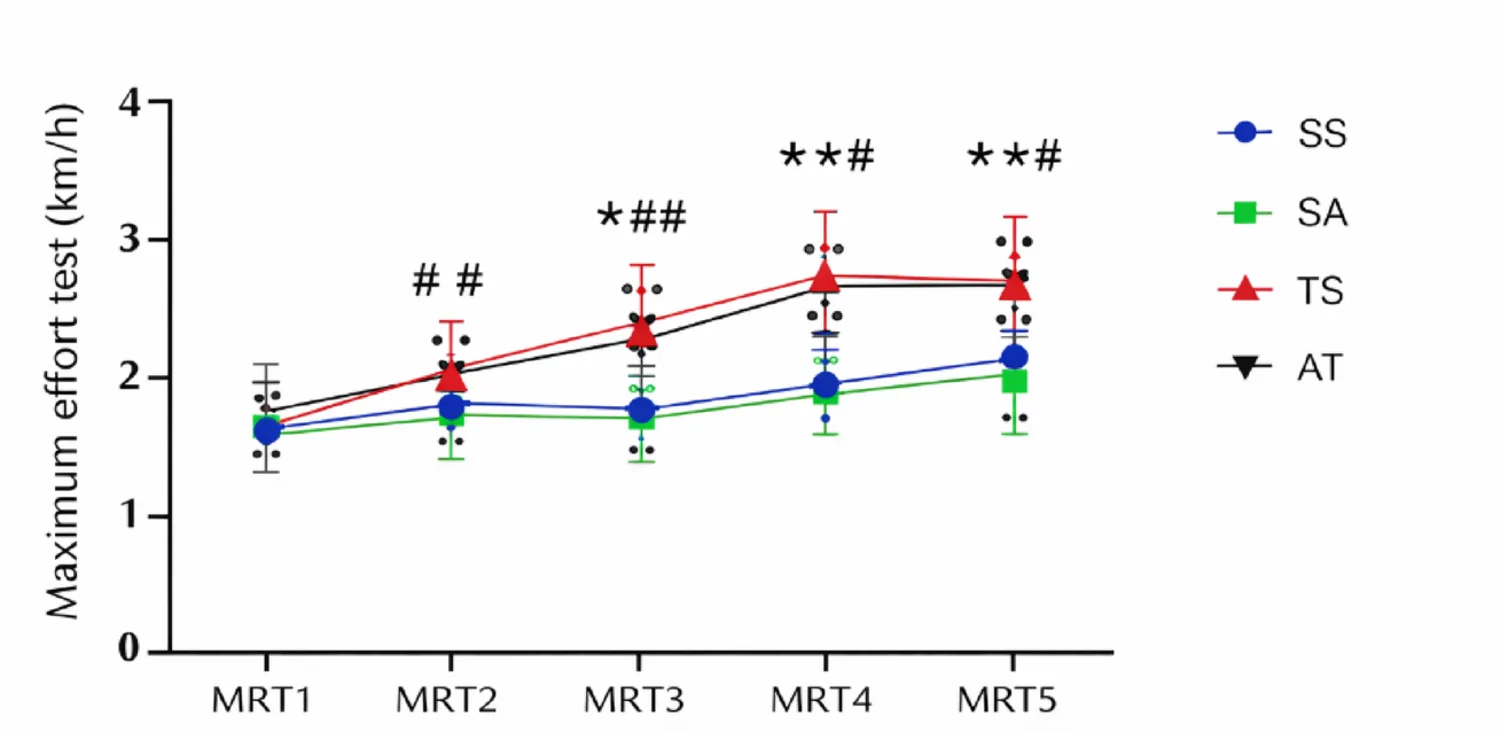
Effect of HIIT on maximal running performance during the experimental period. Maximal running speed (km/h) was assessed in sham sedentary (SS), Alzheimer sedentary (AS), sham trained (ST), and Alzheimer trained (AT) groups across five maximal running tests (MRT1–MRT5). Trained groups showed progressive increases in maximal running speed throughout the intervention, whereas sedentary groups exhibited no substantial improvement over time. Data are presented as mean ± SD. Two-way ANOVA revealed significant effects of group, time, and interaction (*p* < 0.0001 for all factors). ** p < 0.01 vs. SS and AS; #* p* < 0.05 vs. corresponding previous MRT within the same trained group. SS = sham sedentary; AS = Alzheimer sedentary; ST = sham trained; AT = Alzheimer trained

The Maximal Running Test (MRT) was performed periodically throughout the intervention to monitor exercise capacity and adjust training intensity (Fig. 7).

At baseline, no significant differences in maximal running capacity were observed among the experimental groups. Throughout the intervention, both trained groups exhibited progressive increases in maximal running speed, whereas sedentary animals maintained similar performance across evaluations.

Two-way ANOVA revealed significant effects of group, time, and group × time interaction (*p* < 0.0001 for all comparisons).

HIIT significantly increased maximal running capacity in trained groups (*p* < 0.0001).

### Hippocampal histology

Histological evaluation of hippocampal sections was performed to assess neuronal apoptosis in response to β-amyloid infusion and high-intensity interval training (HIIT). Apoptotic neurons were identified based on classical morphological criteria, including condensed chromatin and reduced nuclear volume, as previously described (Puigserver and Spiegelman 2003).

#### Representative histology

**Fig. 8:**
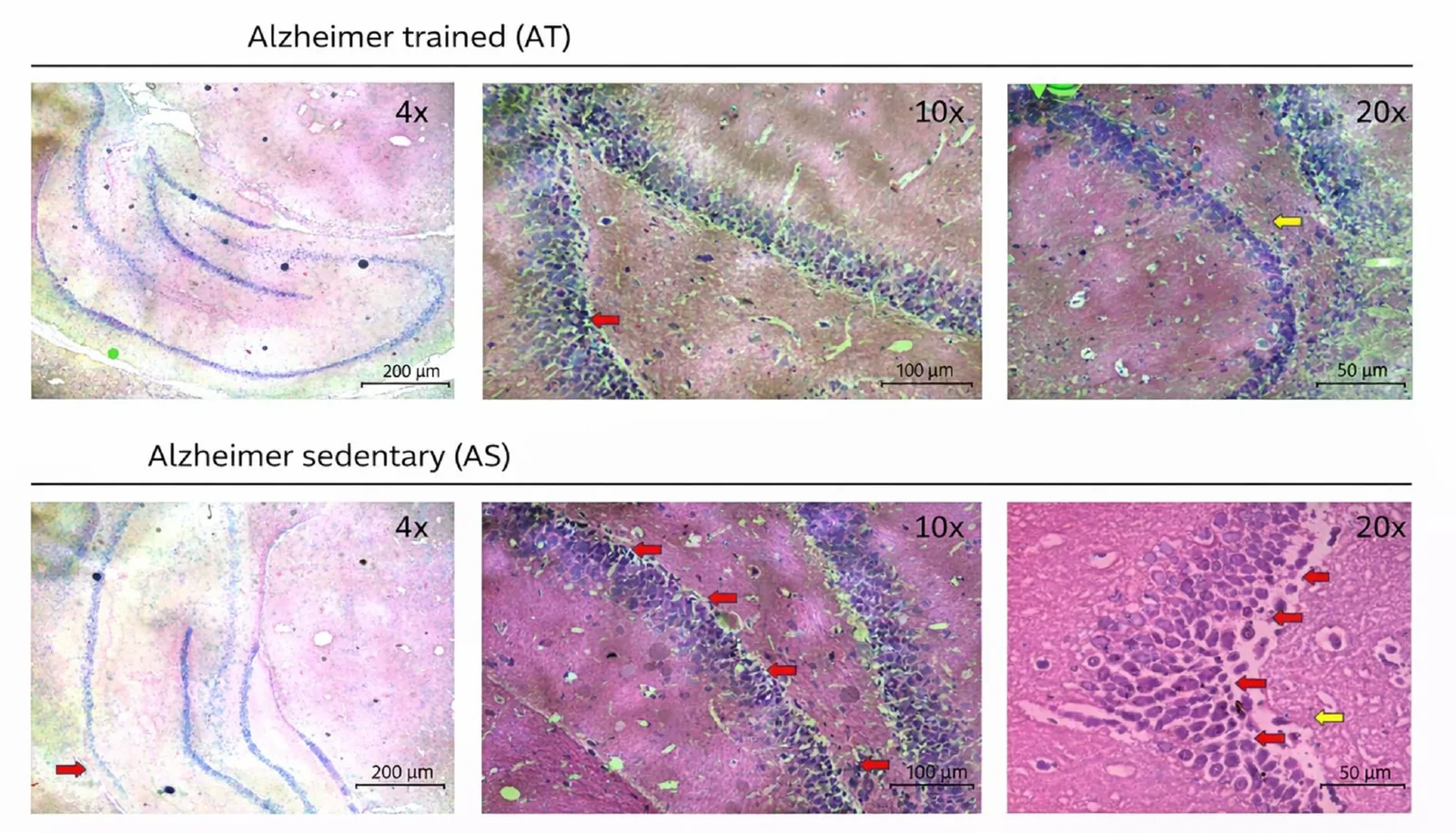
Representative hippocampal histology showing apoptotic neurons in dentate gyrus and CA4 regions. Representative photomicrographs of hippocampal sections from Alzheimer trained (AT, top row) and Alzheimer sedentary (AS, bottom row) animals at increasing magnifications (A = low magnification, B = intermediate magnification, C = high magnification). Red arrows indicate apoptotic neurons characterized by condensed nuclei; yellow arrows indicate preserved neuronal morphology. Alzheimer sedentary animals show increased apoptotic density, whereas trained animals exhibit reduced apoptotic profiles and better preservation of hippocampal cytoarchitecture

Representative photomicrographs showed a higher density of apoptotic neurons in AS animals than in the remaining groups. Trained animals exhibited better preservation of hippocampal cytoarchitecture (Fig. 8).

#### Quantification of apoptotic neurons

**Fig. 9:**
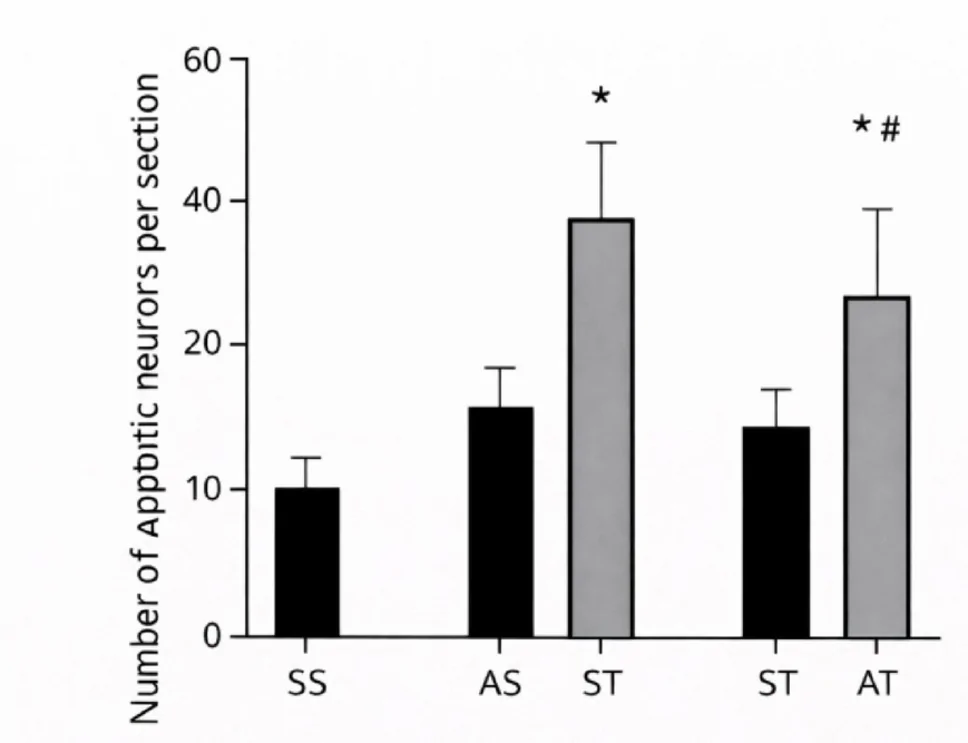
Quantification of apoptotic neurons in hippocampal sections. Mean number of apoptotic neurons per histological section in sham sedentary (SS), Alzheimer sedentary (AS), sham trained (ST), and Alzheimer trained (AT) groups. Data are presented as mean ± SD. Two-way ANOVA revealed significant effects of interaction (*p* = 0.0177), pathological condition (*p* = 0.0001), and training (*p* < 0.0001), followed by Tukey’s post hoc test. * p < 0.001 vs. corresponding sham group; #* p* < 0.05 vs. Alzheimer sedentary group. SS = sham sedentary; AS = Alzheimer sedentary; ST = sham trained; AT = Alzheimer trained

Quantitative analysis was performed using simple random sampling of 10 histological sections per group, according to West (1993). Data were analyzed by two-way ANOVA, which revealed significant effects of interaction (*p* = 0.0177), pathological condition (row factor, *p* = 0.0001), and training (column factor, *p* < 0.0001), indicating that both β-amyloid exposure and exercise significantly influenced apoptotic cell counts (Fig. 9).

The mean number of apoptotic neurons (mean ± SD) was: sham sedentary (SS), 18.10 ± 4.10; sham trained (ST), 14.40 ± 4.68; Alzheimer sedentary (AS), 46.50 ± 11.20; and Alzheimer trained (AT), 32.60 ± 2.72.

Post hoc Tukey analysis demonstrated that Alzheimer sedentary animals presented significantly more apoptotic neurons than sham controls (*p* < 0.001).

HIIT significantly reduced apoptotic neurons in Alzheimer animals (AT vs. AS, *p* < 0.05).

### Gene expression

**Fig. 10:**
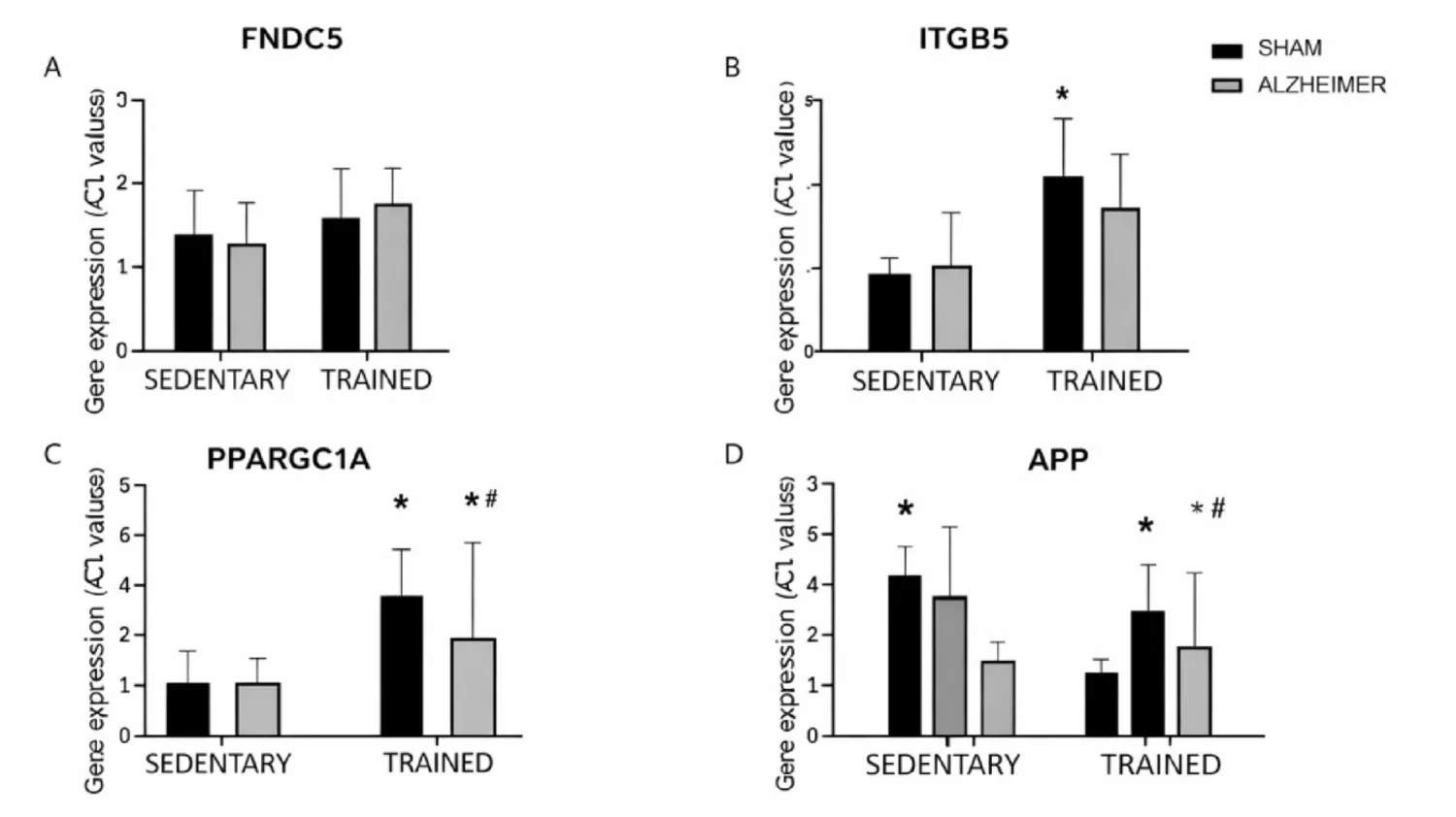
Gene expression analysis of FNDC5, ITGB5, PPARGC1A, and APP in hippocampal tissue. Relative gene expression (ΔCt values) of FNDC5 (A), ITGB5 (B), PPARGC1A (C), and APP (D) in sham sedentary (SS), Alzheimer sedentary (AS), sham trained (ST), and Alzheimer trained (AT) groups. Data are presented as mean ± SD. Two-way ANOVA followed by Tukey’s post hoc test was used for statistical analysis. * p < 0.05 vs. corresponding sham sedentary group; #* p* < 0.05 vs. Alzheimer sedentary group. No significant differences were observed for FNDC5 expression

Relative hippocampal mRNA expression of FNDC5, ITGB5, PPARGC1A, and APP was evaluated to investigate the molecular adaptations associated with high-intensity interval training (HIIT) (Fig. 10).

No significant differences in FNDC5 mRNA expression were observed among the experimental groups (*p* > 0.05). In contrast, ITGB5 expression was significantly higher in the sham-trained group than in the sham sedentary group (*p* < 0.0001), whereas no significant differences were detected between the Alzheimer groups.

Similarly, PPARGC1A expression was significantly increased in trained animals. Both the sham-trained and Alzheimer-trained groups exhibited higher expression levels than their respective sedentary controls (*p* < 0.05), and the Alzheimer-trained group also showed significantly higher expression than the Alzheimer sedentary group (*p* < 0.01).

Finally, APP mRNA expression was significantly reduced following HIIT under both sham and Alzheimer conditions, with lower expression observed in the sham-trained group compared with the sham sedentary group (*p* = 0.0160) and in the Alzheimer trained group compared with the Alzheimer sedentary group (*p* = 0.0390).

### Protein expression

**Fig. 11:**
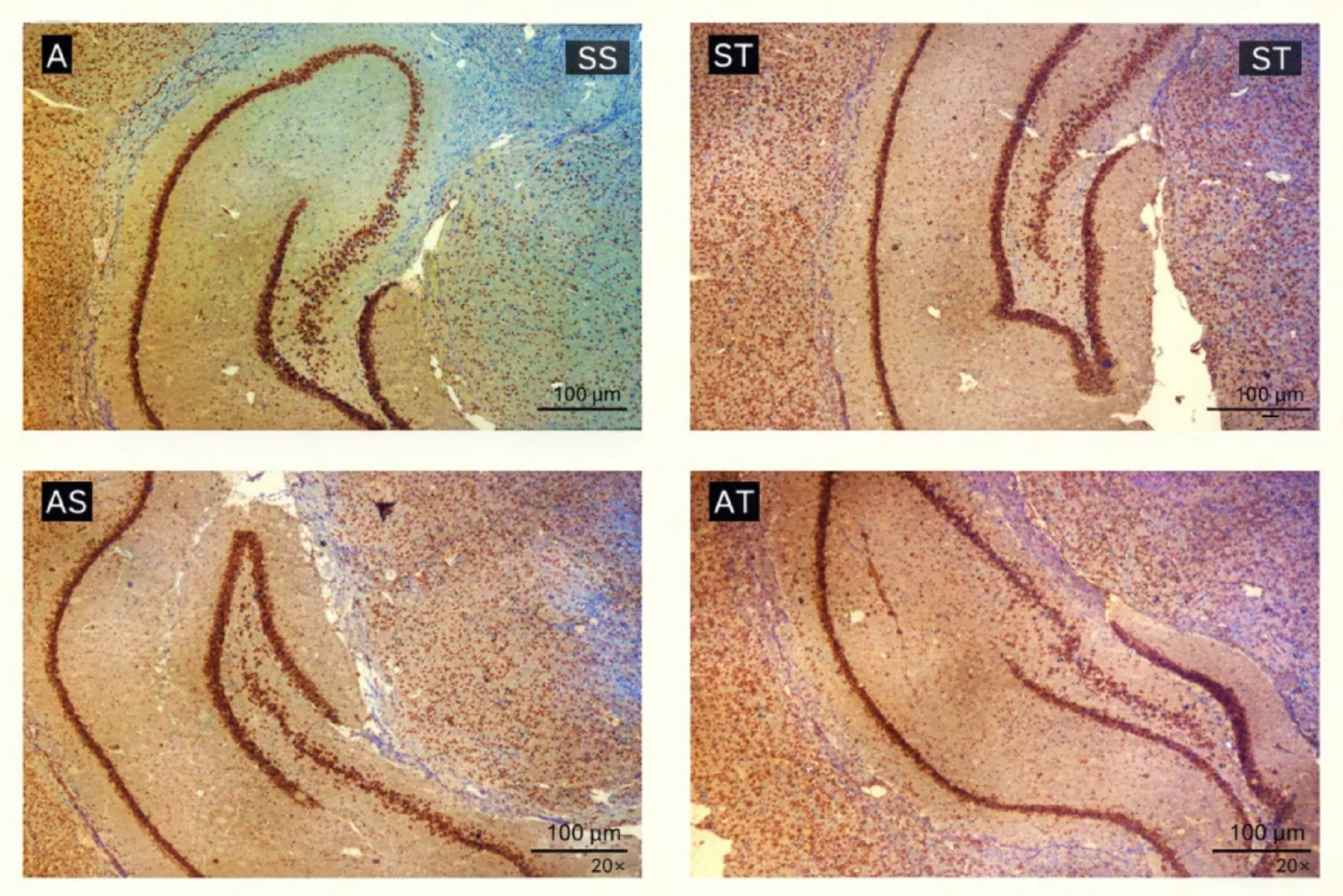
Immunohistochemical detection of FNDC5 in hippocampal sections. Representative photomicrographs of FNDC5 immunoreactivity in the hippocampus (dentate gyrus region) of sham sedentary (SS, A), sham trained (ST, B), Alzheimer sedentary (AS, C), and Alzheimer trained (AT, D) groups. Sections were analyzed at 20× magnification. FNDC5-positive cells are indicated by brown staining. Trained groups exhibit increased immunoreactivity compared to sedentary controls, whereas Alzheimer sedentary animals show reduced staining intensity. These semi-quantitative observations are consistent with the quantitative analysis presented in Fig. 12

FNDC5 immunohistochemistry revealed increased protein expression in trained groups (*p* < 0.0001), independent of pathological condition (Fig. 11).

#### Protein analysis—FNDC5 immunohistochemistry quantification

**Fig. 12:**
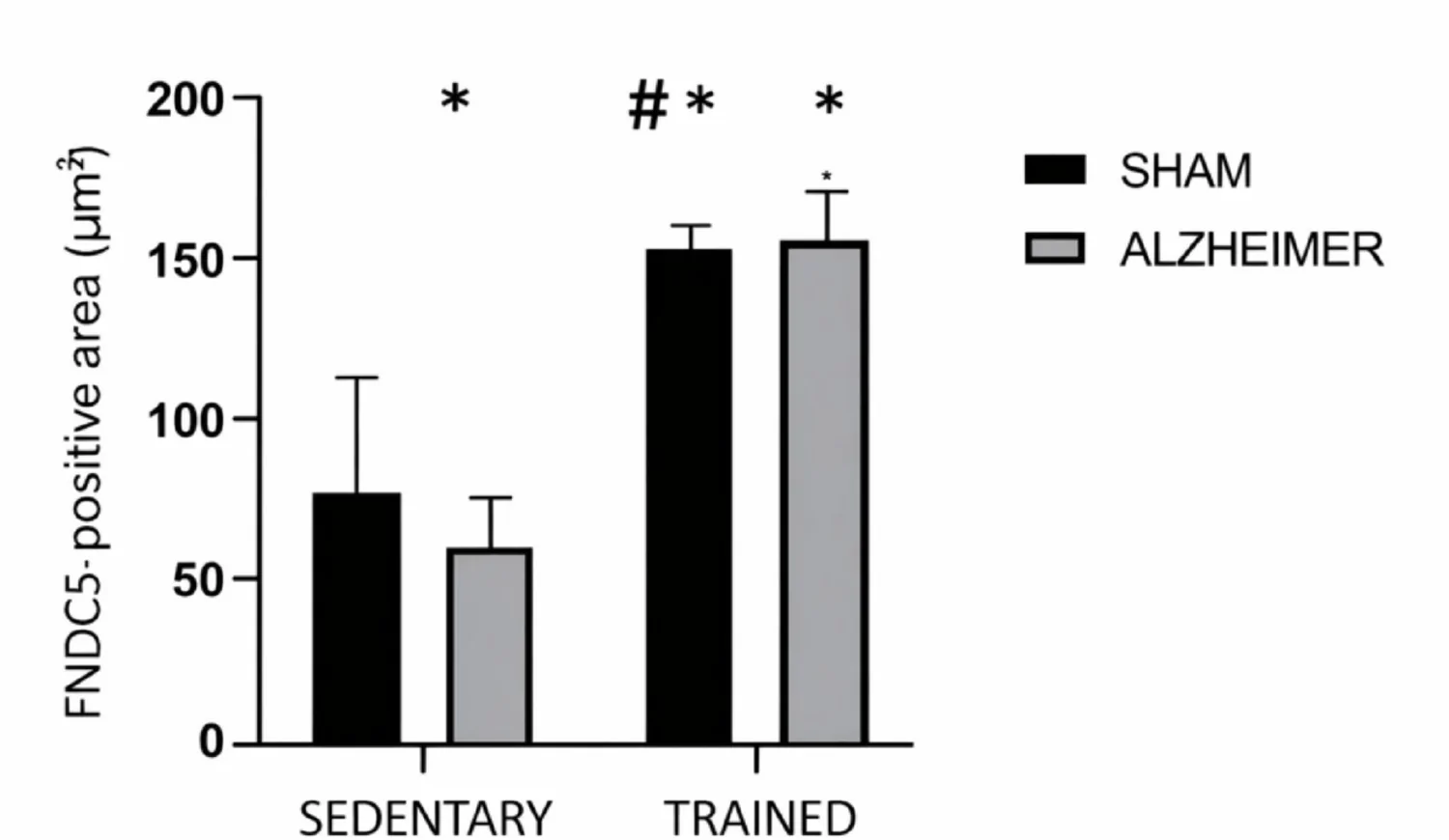
Quantification of FNDC5 immunoreactive area in hippocampal sections. Mean FNDC5-positive area (µm^2^) in sham sedentary (SS), Alzheimer sedentary (AS), sham trained (ST), and Alzheimer trained (AT) groups. Data are presented as mean ± SD. Two-way ANOVA revealed a significant effect of training (*p* < 0.0001), with no significant effect of pathological condition or interaction. * p < 0.01 vs. sham sedentary group; #* p* < 0.01 vs. Alzheimer sedentary group

Quantification of FNDC5 immunoreactive area is presented in Fig. 12. The mean FNDC5-positive area (µm^2^ ± SD) was as follows: sham sedentary (SS), 79.55 ± 37.43; Alzheimer sedentary (AS), 57.76 ± 15.45; sham trained (ST), 155.21 ± 4.96; and Alzheimer trained (AT), 155.30 ± 14.53.

**Fig. 13:**
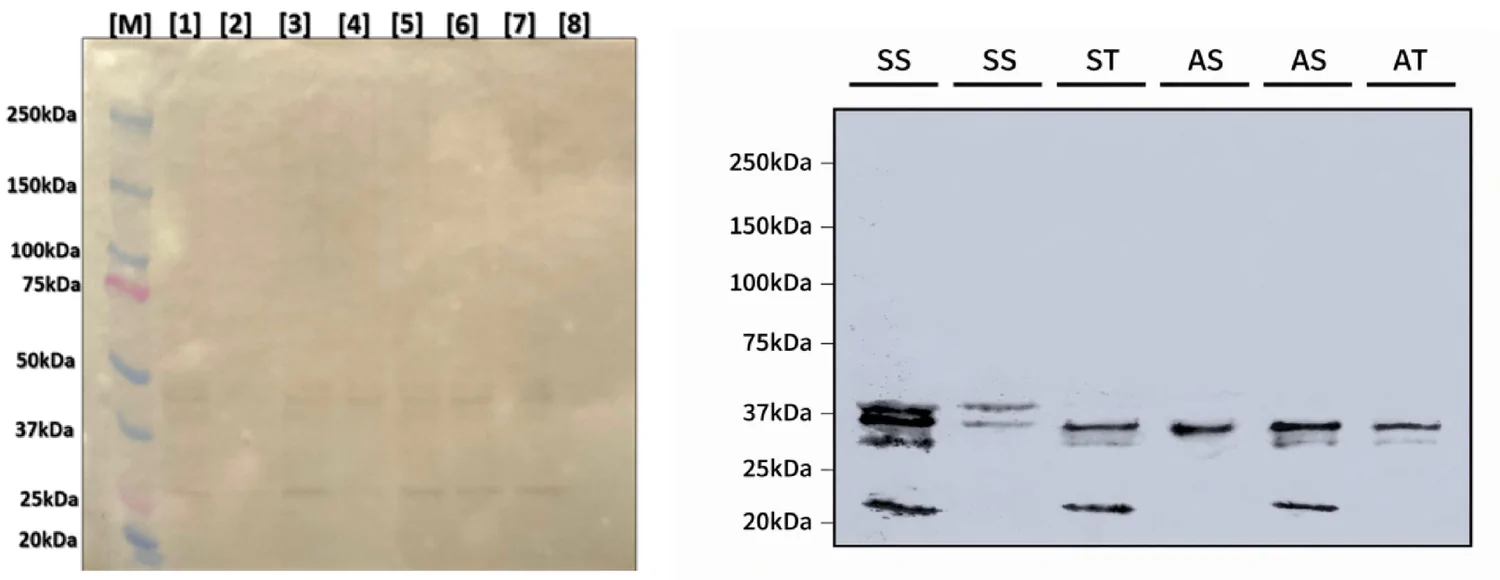
Western blot analysis of APP protein expression in hippocampal tissue. Representative immunoblot showing APP protein bands in sham sedentary (SS), sham trained (ST), Alzheimer sedentary (AS), and Alzheimer trained (AT) groups. Bands are observed at approximately 37 kDa and 25 kDa, (M) Molecular weight marker; lanes (1–2) SS, (3–4) ST, (5–6) AS, and (7–8) AT. Equal protein loading was applied across all samples

Two-way ANOVA revealed a significant effect of training (*p* < 0.0001), whereas no significant effects were observed for pathological condition (*p* = 0.2340) or interaction (*p* = 0.2305). Post hoc analysis demonstrated that both trained groups (ST and AT) exhibited significantly higher FNDC5 immunoreactive area compared to the sham sedentary group (*p* < 0.01). Additionally, the Alzheimer-trained group (AT) showed significantly higher values compared to the Alzheimer sedentary group (*p* < 0.01) (Fig. 13).

#### Protein analysis—densitometric quantification of APP (western blot)

Densitometric quantification revealed distinct patterns of APP protein expression among the experimental groups. Mean APP protein levels (± SD) were 13.68 ± 4.06 in the sham sedentary (SS) group, 3.59 ± 2.29 in the Alzheimer sedentary (AS) group, 11.71 ± 4.14 in the sham trained (ST) group, and 7.24 ± 2.98 in the Alzheimer trained (AT) group.

Two-way ANOVA demonstrated significant effects of pathological condition, training, and their interaction (*p* < 0.0001 for all comparisons). Post hoc analysis showed that APP protein expression was significantly lower in the Alzheimer sedentary group than in the sham sedentary group (*p* < 0.01). In addition, both trained groups differed significantly from the sham sedentary group (*p* < 0.01), whereas APP protein levels were significantly higher in the Alzheimer-trained group than in the Alzheimer sedentary group (*p* < 0.01).

**Fig. 14:**
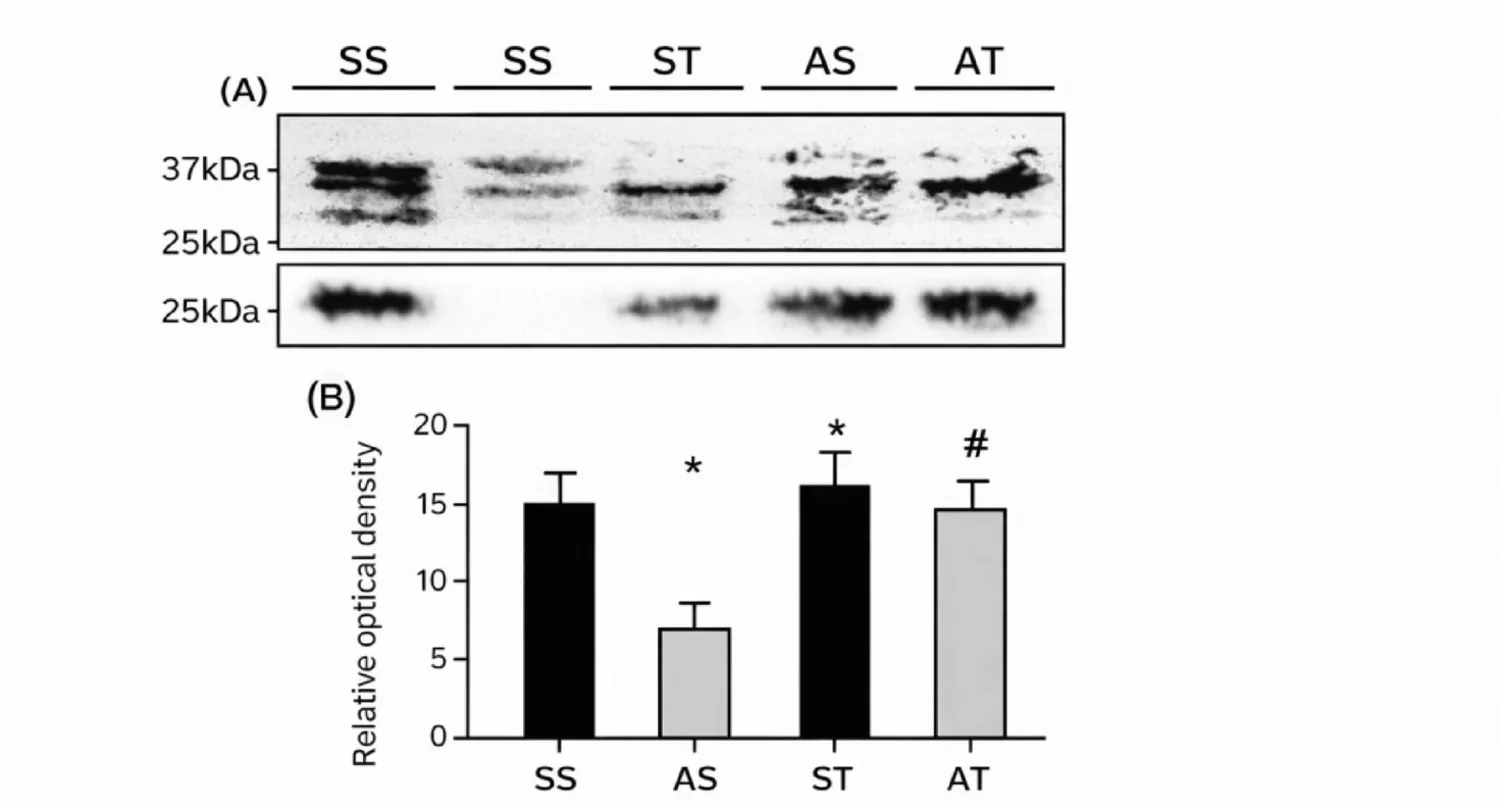
Densitometric quantification of APP protein expression by Western blot. Relative optical density (arbitrary units, a.u.) of APP protein bands in hippocampal tissue from sham sedentary (SS), Alzheimer sedentary (AS), sham trained (ST), and Alzheimer trained (AT) groups. Data are presented as mean ± SD. Two-way ANOVA revealed significant effects of interaction, pathological condition, and training (*p* < 0.0001 for all factors), followed by Tukey’s post hoc test. * p < 0.01 vs. SS; #* p* < 0.01 vs. AS. Representative Western blot images include an internal technical control lane used for quality assurance during membrane processing. This lane was not included in the quantitative analyses

Densitometric quantification of APP protein expression is shown in Fig. 14: Sham Sedentary (SS)—13.68±4.06, Alzheimer Sedentary (AS)—3.59±2.29, Sham Trained (ST)—11.71±4.14, and Alzheimer Trained (AT)—7.24±2.98. The interaction factor was 12.69% with a *p*-value < 0.0001, the row factor was 1.142% with a *p*-value < 0.0001, and the column factor was 85.52% with a *p*-value < 0.0001. Significant differences (*p* < 0.01) were observed between the AS, ST, and AT groups compared to the SS control group (*), and the HIIT training showed differences (*p* < 0.01) between the AT and AS groups (#). No significant reduction in total APP protein was observed despite the transcriptional changes. Representative Western blot images may include an internal technical control lane used to verify consistency between electrophoretic runs and membrane processing. This control lane was not part of the experimental groups and was not included in the densitometric or statistical analyses. Only the APP immunoreactive band corresponding to the expected molecular weight was quantified and normalized to β-actin.

## Discussion

The present study shows that high-intensity interval training (HIIT) was associated with molecular, histological, and physiological adaptations in a β-amyloid-induced rat model. HIIT increased hippocampal PPARGC1A expression, increased FNDC5 protein abundance despite unchanged FNDC5 mRNA, reduced APP transcription, and attenuated neuronal apoptosis. Spatial-memory improvement was limited to a within-group change in trained β-amyloid-exposed animals, without significant between-group differences. These findings therefore support a neuroprotective association but do not establish a causal PGC-1α/FNDC5/APP pathway or robust cognitive recovery.

The growing interest in exercise as a disease-modifying strategy for Alzheimer's disease stems from the recognition that neurodegeneration results from the interaction of multiple pathological processes rather than from amyloid accumulation alone. Mitochondrial dysfunction, oxidative stress, impaired cellular bioenergetics, neuroinflammation, and progressive synaptic failure establish a self-perpetuating cycle that culminates in neuronal death and cognitive decline. Consequently, interventions capable of simultaneously influencing several of these mechanisms are considered particularly attractive because they may exert broader biological effects than therapies directed toward a single molecular target. Within this context, HIIT has emerged as an exercise modality capable of inducing robust systemic and cerebral metabolic adaptations while requiring a relatively low training volume, making it an increasingly relevant intervention in translational neuroscience research (Cummings et al. 2024; Liegro et al. 2023; Swerdlow 2018; Tiwari et al. 2019; Paxinos and Watson 2014; Long and Holtzman 2019; Hampel et al. 2021).

An important aspect of the present findings is that the observed molecular adaptations were accompanied by clear evidence of physiological responsiveness to the training protocol. The progressive improvement in maximal running performance throughout the intervention confirms that the exercise stimulus was sufficient to induce systemic adaptations and reinforces the internal validity of the experimental design. This observation is particularly relevant because many exercise-induced neurobiological responses depend on achieving an adequate metabolic stimulus capable of activating intracellular signaling pathways associated with mitochondrial remodeling and cellular adaptation. Therefore, the physiological improvements observed in the trained animals provide an essential biological framework for interpreting the hippocampal molecular changes identified in the present study (Cotman and Berchtold 2002; Kobilo et al. 2011; Handschin and Spiegelman 2008; St-Pierre et al. 2006).

Although behavioral improvements were modest, the reduction in hippocampal neuronal apoptosis represents one of the most biologically relevant findings of this investigation. Neuronal apoptosis is considered a downstream consequence of several pathological events that characterize Alzheimer's disease, including mitochondrial dysfunction, oxidative stress, calcium dysregulation, excitotoxicity, and chronic neuroinflammation. Consequently, attenuation of apoptotic cell death may reflect the cumulative effect of upstream protective mechanisms rather than an isolated anti-apoptotic action of exercise. This interpretation is consistent with experimental studies demonstrating that physical exercise reduces oxidative damage, preserves mitochondrial integrity, attenuates inflammatory signaling, and promotes neuronal survival even in situations where behavioral recovery remains incomplete (Tiwari et al. 2019; Wang et al. 2020; Cenini and Voos 2019; Lin and Beal 2006; Swerdlow 2018, 2007; Liegro et al. 2023; Erickson et al. 2019; Voss et al. 2013; Sleiman et al. 2016; Wrann 2015; Lourenco and Frozza 2023; Pedersen 2019; Hardy and Selkoe 2002).

The apparent discrepancy between the robust molecular and histological adaptations and the limited behavioral improvement should also be interpreted cautiously. Cognitive performance in experimental models of Alzheimer's disease is influenced by multiple variables, including the severity of β-amyloid pathology, the duration of the intervention, the sensitivity of behavioral paradigms, and the temporal relationship between molecular recovery and functional restoration. Increasing evidence suggests that exercise-induced adaptations frequently emerge first at the molecular and cellular levels, with measurable cognitive benefits becoming evident only after sustained preservation of neuronal circuits. Therefore, the absence of significant between-group differences in the Morris water maze does not diminish the biological relevance of the molecular findings but rather supports the concept that neuroprotection may precede detectable behavioral recovery (Cotman and Berchtold 2002; Puigserver and Spiegelman 2003; Paxinos and Watson 2014; Hampel et al. 2021; Voss et al. 2013; Sleiman et al. 2016; Wrann 2015; Lourenco and Frozza 2023).

The increase in PPARGC1A expression provides a plausible interpretive framework for relating exercise-induced physiological adaptation to the observed changes in FNDC5, APP transcription, and neuronal survival. However, pathway activation and causal ordering were not directly tested in the present study.

The significant increase in hippocampal PPARGC1A expression observed following HIIT represents one of the central findings of the present study and provides an important mechanistic framework for interpreting the remaining molecular adaptations. PGC-1α has emerged as a master regulator of cellular energy metabolism, coordinating transcriptional programs involved in mitochondrial biogenesis, oxidative phosphorylation, antioxidant defense, and metabolic flexibility. Although initially characterized in skeletal muscle, increasing evidence demonstrates that PGC-1α also plays a critical role within the central nervous system, where it contributes to neuronal energy homeostasis, synaptic maintenance, and resistance to oxidative injury (Handschin and Spiegelman 2008; St-Pierre et al. 2006; Katsouri et al. 2011; Boström et al. 2012; Voss et al. 2013).

The biological significance of this finding extends beyond mitochondrial biogenesis alone. Neurons are among the most metabolically demanding cells in the body, relying almost exclusively on oxidative metabolism to sustain membrane excitability, neurotransmitter recycling, axonal transport, and synaptic transmission. Consequently, even subtle disturbances in mitochondrial function may compromise neuronal viability. In Alzheimer's disease, mitochondrial dysfunction is now recognized as an early pathogenic event that precedes extensive neuronal loss, contributing to ATP depletion, excessive reactive oxygen species production, calcium dyshomeostasis, and activation of intrinsic apoptotic pathways. Restoration of mitochondrial function has therefore become an important therapeutic objective in contemporary Alzheimer's disease research (Swerdlow 2018; Tiwari et al. 2019; Wang et al. 2020; Cenini and Voos 2019; Lin and Beal 2006; Liegro et al. 2023; Erickson et al. 2019; Hampel et al. 2021; Querfurth and LaFerla 2010; Mattson 2012).

Within this context, the increase in PPARGC1A expression observed after HIIT is compatible with an adaptive metabolic response to β-amyloid-induced stress. Because mitochondrial function and downstream PGC-1α activity were not directly measured, this finding should be interpreted as transcriptional evidence rather than proof of enhanced mitochondrial resilience.

Another important aspect concerns the relationship between PGC-1α and amyloid pathology. Experimental evidence indicates that PGC-1α negatively regulates the transcription of BACE1, the β-secretase responsible for initiating the amyloidogenic processing of APP. Reduced BACE1 expression decreases the generation of amyloid-β peptides, providing a mechanistic link between exercise-induced metabolic adaptation and modulation of Alzheimer's disease pathology. Although BACE1 activity was not evaluated in the present investigation, the simultaneous increase in PPARGC1A expression and reduction in APP transcription observed in trained animals is consistent with this regulatory model. These findings reinforce the hypothesis that HIIT influences amyloid-related pathways indirectly through metabolic remodeling rather than through direct interference with APP itself (Boström et al. 2012; Hampel et al. 2021; Querfurth and LaFerla 2010; Mattson 2012; Wilson et al. 2023).

The present findings also support the concept that the neuroprotective effects of HIIT should be interpreted as systemic biological adaptations rather than isolated molecular responses. High-intensity interval exercise imposes repeated energetic challenges that stimulate intracellular signaling pathways associated with AMPK activation, calcium-dependent kinases, and sirtuin-mediated regulation, all of which converge on PGC-1α activation. Once activated, PGC-1α orchestrates a broad transcriptional program that extends beyond energy metabolism, influencing inflammatory regulation, oxidative stress responses, mitochondrial dynamics, and neuronal survival. Consequently, the increase in PPARGC1A expression identified in this study is unlikely to represent an isolated event, but instead appears to reflect activation of a coordinated adaptive program initiated by repeated metabolic stimulation during HIIT (Kobilo et al. 2011; Handschin and Spiegelman 2008; St-Pierre et al. 2006; Katsouri et al. 2011; Voss et al. 2013; Sleiman et al. 2016; Wrann 2015; Lourenco and Frozza 2023; Pedersen 2019; Swerdlow 2007; Hardy and Selkoe 2002).

The convergence of improved exercise capacity, altered molecular markers, and reduced neuronal apoptosis is compatible with a role for PGC-1α-related metabolic regulation in the observed neurobiological response. Nevertheless, the present design cannot determine whether PPARGC1A change was upstream of FNDC5, APP transcription, or neuronal apoptosis.

Taken together, the findings are compatible with, but do not demonstrate, a model in which HIIT-related metabolic adaptation involves PPARGC1A and FNDC5 and occurs alongside changes in APP transcription and neuronal apoptosis. Direct pathway measurements or experimental manipulation of these targets will be required to test this sequence.

One of the most intriguing findings of the present study was the apparent dissociation between FNDC5 gene and protein expression. Whereas hippocampal FNDC5 mRNA levels remained unchanged following HIIT, FNDC5 protein abundance increased significantly in both trained groups, irrespective of pathological condition. Rather than representing conflicting observations, this pattern illustrates the complexity of post-transcriptional regulation and emphasizes that transcriptional activity alone does not necessarily predict final protein abundance. In complex biological systems, particularly in response to physiological stimuli such as exercise, protein expression is influenced by multiple regulatory mechanisms acting after gene transcription, including mRNA stability, translational efficiency, protein degradation, intracellular trafficking, and post-translational modifications (Wrann et al. 2013; Lourenco et al. 2019; Kim et al. 2018; Islam et al. 2024; Falck et al. 2024; Paxinos and Watson 2014; Pedersen 2019; Swerdlow 2007; Hardy and Selkoe 2002).

These findings reinforce an important methodological consideration for studies investigating exercise-induced molecular adaptations. Reliance exclusively on gene expression analyses may underestimate biologically relevant responses because many adaptive processes occur primarily at the translational or post-translational level. Physical exercise represents a dynamic physiological stimulus capable of rapidly modifying intracellular signaling pathways that regulate protein synthesis and degradation without necessarily producing proportional changes in messenger RNA levels. Therefore, the simultaneous evaluation of transcriptional and protein responses, as performed in the present study, provides a more comprehensive characterization of the molecular adaptations induced by HIIT than either approach alone (Wrann et al. 2013; Lourenco et al. 2019; Kim et al. 2018; Islam et al. 2024; Falck et al. 2024; Paxinos and Watson 2014). The increase in FNDC5 protein observed in trained animals gains additional relevance considering the biological functions currently attributed to the PGC-1α/FNDC5/irisin signaling pathway. FNDC5 was initially described as a membrane protein induced by PGC-1α in skeletal muscle, where its proteolytic cleavage generates irisin, a myokine capable of mediating systemic adaptations to physical exercise. More recently, accumulating evidence has demonstrated that this signaling axis extends beyond peripheral metabolic regulation and participates directly in communication between skeletal muscle and the central nervous system. This concept, currently referred to as the muscle–brain axis, proposes that exercise-induced myokines act as endocrine mediators capable of influencing neuronal metabolism, neuroplasticity, and resistance to neurodegenerative processes (Pedersen and Febbraio 2012; Kobilo et al. 2011; Wrann et al. 2013; Lourenco et al. 2019; Kim et al. 2018; Islam et al. 2024; Falck et al. 2024; Paxinos and Watson 2014; Pedersen 2019; Swerdlow 2007; Hardy and Selkoe 2002).

Experimental studies have shown that activation of the PGC-1α/FNDC5 pathway promotes increased expression of brain-derived neurotrophic factor (BDNF), stimulates hippocampal neurogenesis, enhances synaptic plasticity, attenuates neuroinflammatory responses, and improves neuronal resistance to oxidative injury. Although BDNF and circulating irisin concentrations were not quantified in the present investigation, the increased hippocampal FNDC5 protein expression observed after HIIT is consistent with activation of this neuroprotective signaling cascade. Importantly, recent evidence suggests that the biological effects of irisin are mediated, at least in part, through interaction with αV integrin receptors, including integrin β5 (ITGB5), which has been identified as one of its principal receptors in several tissues. This observation provides an important conceptual framework for interpreting the simultaneous evaluation of FNDC5 and ITGB5 performed in the present study (Lourenco et al. 2019; Kim et al. 2018; Islam et al. 2024; Falck et al. 2024; Paxinos and Watson 2014; Pedersen 2019; Swerdlow 2007; Hardy and Selkoe 2002).

Interestingly, although ITGB5 expression increased significantly only in the sham-trained group, no comparable increase was observed under Alzheimer's disease conditions. This finding may indicate that β-amyloid pathology partially impairs the responsiveness of the FNDC5/ITGB5 signaling axis, even in the presence of an adequate exercise stimulus. Alternatively, it is possible that receptor regulation differs from ligand regulation, involving distinct temporal dynamics or compensatory mechanisms that were not captured at the single experimental time point evaluated in this study. Since receptor activation depends not only on transcription but also on membrane localization, receptor recycling, ligand availability, and downstream signaling efficiency, interpretation of ITGB5 expression should be made cautiously. Additional investigations evaluating receptor protein abundance and signaling activity will be necessary to clarify the functional significance of these observations (Islam et al. 2024; Falck et al. 2024).

The combined increase in PPARGC1A expression and FNDC5 protein is consistent with coordinated exercise-responsive adaptation. However, these parallel changes do not demonstrate that PGC-1α induced FNDC5 or that either change mediated the effects on APP transcription or neuronal apoptosis.

If the PGC-1α/FNDC5 axis contributes to the response to HIIT, modulation of APP-related biology and neuronal survival could represent downstream consequences. This remains a hypothesis because irisin, receptor activation, BACE1, APP processing, and pathway mediation were not directly assessed.

Among the molecular adaptations observed following HIIT, the reduction in hippocampal APP gene expression represents one of the most relevant findings from a pathophysiological perspective. Although the amyloid cascade hypothesis has evolved substantially over the past decades, abnormal APP metabolism remains a central event in Alzheimer's disease because it provides the substrate for the generation of amyloid-β peptides and initiates a cascade of synaptic dysfunction, mitochondrial impairment, neuroinflammation, and neuronal degeneration. Consequently, interventions capable of modulating APP expression or processing continue to attract considerable interest as potential disease-modifying strategies (Bertram et al. 2010; Selkoe and Hardy 2016; Liegro et al. 2023; Tiwari et al. 2019; Hampel et al. 2021; Querfurth and LaFerla 2010; Mattson 2012; Gomez-Pinilla and Hillman 2013).

The present findings demonstrate that HIIT significantly reduced APP transcription in animals exposed to β-amyloid while simultaneously attenuating hippocampal neuronal apoptosis. Although the present experimental design does not allow the establishment of direct mechanistic relationships between these events, their concomitant occurrence is consistent with the hypothesis that exercise-induced metabolic adaptations create a cellular environment less favorable for amyloid-associated neurotoxicity. Rather than directly suppressing APP expression through a single signaling pathway, HIIT may influence multiple upstream regulatory mechanisms that collectively reduce amyloidogenic pressure within the hippocampus. This interpretation aligns with the contemporary understanding that Alzheimer's disease arises from interacting metabolic and cellular disturbances rather than from isolated abnormalities in amyloid metabolism (Cummings et al. 2024; Bertram et al. 2010; Selkoe and Hardy 2016; Liegro et al. 2023; Swerdlow 2018; Tiwari et al. 2019; Hampel et al. 2021; Querfurth and LaFerla 2010; Mattson 2012; Gomez-Pinilla and Hillman 2013; Voss et al. 2013).

An important aspect of this interpretation is the previously discussed activation of the PPARGC1A pathway. Experimental studies have demonstrated that PGC-1α negatively regulates the expression of BACE1, the β-secretase responsible for initiating the amyloidogenic cleavage of APP. Reduced BACE1 activity decreases amyloid-β generation and has been associated with attenuation of plaque formation and neuronal injury in experimental models of Alzheimer's disease. Although neither BACE1 expression nor secretase activity was evaluated in the present study, the simultaneous increase in PPARGC1A and reduction in APP expression observed following HIIT are compatible with this mechanistic framework. Accordingly, the present findings support the concept that metabolic remodeling induced by exercise may indirectly influence amyloid-related pathways through coordinated regulation of upstream cellular metabolism (Boström et al. 2012; Hampel et al. 2021; Querfurth and LaFerla 2010; Mattson 2012).

The reduction in APP transcription also acquires additional significance when interpreted together with the histological findings. Excessive amyloidogenic signaling contributes to mitochondrial dysfunction, oxidative stress, disruption of intracellular calcium homeostasis, and activation of intrinsic apoptotic pathways. Therefore, the lower number of apoptotic neurons observed in trained Alzheimer's animals may represent one downstream consequence of reduced amyloid-associated cellular stress. This interpretation is supported by experimental evidence demonstrating that exercise attenuates oxidative damage, improves mitochondrial function, reduces neuroinflammatory signaling, and preserves neuronal integrity even in experimental models where complete reversal of cognitive deficits is not achieved. Rather than functioning through a single anti-apoptotic mechanism, HIIT appears to promote a broader neuroprotective environment that increases neuronal resistance to β-amyloid toxicity (Tiwari et al. 2019; Wang et al. 2020; Cenini and Voos 2019; Lin and Beal 2006; Swerdlow 2018, 2007; Liegro et al. 2023; Erickson et al. 2019; Voss et al. 2013; Sleiman et al. 2016; Wrann 2015; Lourenco and Frozza 2023; Pedersen 2019; Hardy and Selkoe 2002).

Interestingly, despite the reduction in APP gene expression, APP protein abundance assessed by Western blot did not differ significantly among experimental groups. At first glance, this apparent discrepancy might suggest an inconsistency between transcriptional and protein findings. However, such divergence is biologically plausible and reflects the complex regulation of APP metabolism. Protein abundance depends not only on transcriptional activity but also on translational efficiency, protein turnover, intracellular trafficking, and enzymatic processing by α-, β-, and γ-secretases. Consequently, changes in APP mRNA may precede detectable alterations in total protein content, particularly during relatively short intervention periods. Moreover, total APP protein does not necessarily reflect the generation of amyloidogenic fragments, which depends primarily on post-translational processing rather than on absolute protein abundance. Therefore, the absence of differences in APP protein should not be interpreted as evidence against biological modulation but rather as an indication that transcriptional regulation and protein processing may occur with distinct temporal dynamics (Bertram et al. 2010; Selkoe and Hardy 2016; Liegro et al. 2023; Swerdlow 2018; Tiwari et al. 2019; Wang et al. 2020; Cenini and Voos 2019; Lin and Beal 2006).

This distinction is particularly important because recent Alzheimer's disease research has progressively shifted its focus from absolute amyloid burden toward the broader concept of proteostasis and cellular resilience. Rather than acting solely by reducing the amount of APP available for amyloidogenic processing, exercise may enhance the capacity of neurons to maintain protein homeostasis, preserve mitochondrial quality, and tolerate proteotoxic stress. Within this framework, the reduction in APP transcription observed after HIIT becomes one component of a coordinated adaptive response involving improved metabolic regulation, mitochondrial preservation, and increased resistance to neurodegeneration, rather than an isolated molecular endpoint (Liegro et al. 2023; Swerdlow 2018; Tiwari et al. 2019; Hampel et al. 2021; Querfurth and LaFerla 2010; Mattson 2012; Gomez-Pinilla and Hillman 2013).

Collectively, these observations strengthen the interpretation that modulation of APP represents an integral component of the exercise-induced neuroprotective phenotype identified in the present study. Nevertheless, preservation of molecular and histological integrity does not necessarily translate immediately into complete functional recovery. Cognitive performance depends on the coordinated activity of complex neuronal networks, and restoration of these networks frequently requires longer periods than those necessary for molecular adaptation. Consequently, the modest behavioral improvements observed in the present investigation should be interpreted within the temporal dynamics of neuroplasticity rather than as evidence of limited biological efficacy of HIIT.

Although HIIT induced molecular and histological adaptations, spatial memory improved only within the trained Alzheimer group, and no significant between-group differences were detected. This discrepancy may reflect temporal differences between molecular adaptation and behavioral recovery, but it may also reflect limited sensitivity or limited cognitive impairment in the model. Therefore, the behavioral null findings should not be interpreted as evidence of cognitive efficacy.

The present findings therefore reinforce the concept that molecular biomarkers may provide greater sensitivity than behavioral outcomes for detecting early neuroprotective responses to exercise interventions. Alterations in mitochondrial metabolism, transcriptional regulation, and neuronal survival are likely to precede improvements in learning and memory because preservation of cellular integrity constitutes a prerequisite for subsequent recovery of neural circuit function. Similar dissociations between robust molecular adaptations and delayed behavioral improvement have been reported in previous experimental studies investigating physical exercise in neurodegenerative disease models, supporting the interpretation that neurobiological recovery follows a progressive rather than simultaneous sequence of events (Cotman and Berchtold 2002; Pedersen 2011; Puigserver and Spiegelman 2003; Voss et al. 2013; Sleiman et al. 2016; Wrann 2015; Lourenco and Frozza 2023).

Several limitations should be considered when interpreting the present findings. First, the study employed an experimental β-amyloid-induced rat model, which reproduces important pathological features of Alzheimer's disease but does not fully capture the complexity and chronic progression of the human disorder. Second, the intervention period may not have been sufficiently long to allow complete translation of molecular adaptations into measurable cognitive recovery. Third, although the present investigation evaluated key components of the proposed neuroprotective pathway, including PPARGC1A, FNDC5, ITGB5, APP, and neuronal apoptosis, other relevant mediators such as BDNF, BACE1, inflammatory cytokines, oxidative stress markers, and mitochondrial respiratory function were not directly assessed. Their inclusion would contribute to a more comprehensive characterization of the biological mechanisms underlying the observed adaptations. Finally, quantification of circulating irisin and assessment of APP processing by α-, β-, and γ-secretases would further clarify the mechanistic links between exercise-induced metabolic remodeling and modulation of amyloid-related pathways.

Despite these limitations, the present study possesses several important strengths. By integrating behavioral assessment with gene expression, protein quantification, histological analysis, and physiological evaluation of exercise capacity, this investigation provides a multidimensional characterization of the neurobiological effects of HIIT in experimental Alzheimer's disease. Furthermore, simultaneous evaluation of transcriptional and protein responses allowed identification of biologically relevant dissociations, particularly regarding FNDC5, emphasizing that exercise-induced adaptations cannot be fully understood through transcriptomic analyses alone. The integrated interpretation of PPARGC1A, FNDC5, APP, and neuronal apoptosis also contributes to a more comprehensive understanding of the molecular networks activated by high-intensity exercise than approaches focused on isolated biomarkers.

Future studies should investigate the long-term effects of HIIT on disease progression, evaluate additional components of the PGC-1α/FNDC5/irisin signaling axis, and determine whether exercise-induced metabolic remodeling can potentiate the effects of emerging disease-modifying therapies for Alzheimer's disease. Longitudinal investigations incorporating advanced analyses of mitochondrial function, neuroinflammation, synaptic plasticity, and protein homeostasis will be particularly valuable for establishing the temporal sequence through which exercise-induced molecular adaptations ultimately translate into functional preservation of cognition. Such studies may also help identify biomarkers capable of monitoring therapeutic responsiveness and optimizing exercise prescriptions for individuals at different stages of neurodegeneration.

In summary, HIIT was associated with improved physical performance, increased PPARGC1A expression, increased FNDC5 protein abundance, reduced APP transcription, and attenuated hippocampal neuronal apoptosis. APP protein abundance did not show a uniform training-related reduction, and cognitive findings were limited to a within-group improvement without significant between-group differences. The molecular and histological results are therefore consistent with a neuroprotective response, while the proposed PGC-1α/FNDC5/APP relationship remains an interpretive hypothesis.

Further studies should directly assess pathway activity, APP processing, BACE1 and secretase activity, amyloid burden, and cognitive outcomes in models with a robust behavioral deficit to determine whether these associated adaptations form a causal neuroprotective mechanism.

## Conclusion

The present study demonstrates that high-intensity interval training (HIIT) induces relevant neurobiological adaptations in a β-amyloid-induced model of Alzheimer's disease.

HIIT significantly improved physical performance and reduced neuronal apoptosis in the hippocampus, indicating a protective association against β-amyloid-induced cellular damage. In addition, HIIT reduced APP gene expression, whereas APP protein abundance differed among experimental conditions without a uniform training-related reduction, supporting transcriptional modulation but not a consistent reduction in total APP protein.

Furthermore, HIIT increased PPARGC1A expression, supporting the activation of metabolic and mitochondrial pathways in hippocampal tissue. Although FNDC5 gene expression remained unchanged, increased FNDC5 protein levels suggest post-transcriptional regulation and reinforce its potential involvement in exercise-induced neurobiological adaptations.

Regarding cognitive performance, HIIT promoted improvements within the Alzheimer-trained group; however, no significant differences were observed between experimental groups. Together with the absence of a robust cognitive deficit in the experimental model, these findings indicate that behavioral outcomes should be interpreted with caution.

Overall, the present findings demonstrate that HIIT promotes molecular and cellular adaptations consistent with neuroprotection, particularly through modulation of metabolic pathways, regulation of APP gene expression, increased FNDC5 protein levels, and attenuation of neuronal apoptosis. Under the present experimental conditions, these biological effects were more pronounced than improvements in cognitive performance.

These findings support the potential of HIIT as a non-pharmacological intervention capable of modulating multiple biological pathways associated with Alzheimer's disease pathophysiology. Nevertheless, additional studies using experimental models with more pronounced cognitive impairment and incorporating complementary molecular targets are warranted to further elucidate the mechanisms underlying exercise-induced neuroprotection.

## Data Availability

No datasets were generated or analysed during the current study.
